# Comprehensive Analysis of *ABCG2* Genetic Variation in the Polish Population and Its Inter-Population Comparison

**DOI:** 10.3390/genes11101144

**Published:** 2020-09-29

**Authors:** Marcin Słomka, Marta Sobalska-Kwapis, Małgorzata Korycka-Machała, Jarosław Dziadek, Grzegorz Bartosz, Dominik Strapagiel

**Affiliations:** 1 Biobank Lab, Department of Molecular Biophysics, Faculty of Biology and Environmental Protection, University of Łódź, Pilarskiego 14/16, 90-231 Łódź, Poland; marcin.slomka@biol.uni.lodz.pl (M.S.); marta.sobalska@biol.uni.lodz.pl (M.S.-K.); 2 BBMRI.pl Consortium, 54-066 Wrocław, Poland; 3 Institute of Medical Biology, Polish Academy of Sciences, Lodowa 106, 93-232 Łódź, Poland; mkorycka@cbm.pan.pl (M.K.-M.); jdziadek@cbm.pan.pl (J.D.); 4 Department of Molecular Biophysics, Faculty of Biology and Environmental Protection, University of Łódź, Pomorska 141/143, 90-236 Łódź, Poland; grzegorz.bartosz@biol.uni.lodz.pl

**Keywords:** High Resolution Melting, ABCG2, BCRP, gene scanning, Polish population

## Abstract

ATP-binding cassette sub-family G member 2 (ABCG2), also known as breast cancer resistance protein (BCRP), is one of the key efflux ATP-binding cassette (ABC) transporters of xenobiotics, their metabolites and endogenous compounds such as urate. Some of its genetic variants have been found to influence protein functioning, resulting in serious clinical implications concerning chemotherapy response, as well as gout or blood group phenotype Jr(a-). Previous reports have suggested that the frequencies of certain crucial polymorphisms, such as c.34G>A (p.Val12Met) and c.421C>A (p.Gln141Lys) differ significantly between the Polish population and other Caucasian populations. Thus, to clarify this issue, the present study performs a complete analysis of the genetic variation of *ABCG2* coding sequence in the Polish population. The genetic variation in 14 out of 15 coding exons of the *ABCG2* gene, as well as their flanking intron sequences, were examined among 190 healthy representatives of the Polish population using scanning with High Resolution Melting (HRM). HRM scanning revealed 17 polymorphisms: eight in the exons (including five missense variants and one point-nonsense mutation) and nine in the intron sequences (eight single nucleotide polymorphisms (SNPs) and one deletion variant). These included variants correlating with the presence of gout and phenotype Jr(a-). Linkage disequilibrium, haplotype blocks and haplotype analyses were also performed. The frequencies of the most common polymorphisms in the Polish population did not differ significantly to those observed for other Caucasian populations, but demonstrated divergence from non-Caucasian populations. We hope that our findings may be helpful for other researchers and clinicians, evaluating the pharmacogenetic role of *ABCG2*.

## 1. Introduction

Among the members of the ATP-binding cassette (ABC) transporters family, ABCG2 functions as one of the key efflux transporters of xenobiotics and their metabolites [1]. Due to it being first isolated from multidrug-resistant human breast cancer cells, the transporter has also been given the name “breast cancer resistance protein” (BCRP) [2]. The *ABCG2* gene is located on chromosome 4q22, where it spans over 66 kbp and contains 16 exons encoding a 655 amino acid protein of molecular weight 72.6 kDa [3,4]. ABCG2 is also known as the “half ABC transporter” because has only one ATP-binding cassette and six transmembrane domains, and hence requires dimerization or oligomerization to transport substrates [5].

ABCG2 plays a protective role and hence is widely expressed in normal cells and tissues, such as capillary endothelial cells, gastrointestinal tract, and hematopoietic stem cells, as well as the tissue of the liver, kidney, and testes; it has also been identified in the maternal glands, the maternal–fetal barrier of the placenta, and the blood-brain barrier [1,5]. However, it has been suggested that ABCG2 expression may be associated with a poor response to cancer chemotherapy and clinical drug resistance [6] associated with the efflux of drugs from cancer cells and multidrug resistance (MDR) to a variety of chemotherapeutic agents, small-molecule inhibitors like tyrosine kinase inhibitors (TKIs) or cyclin-dependent kinase 9 (CDK9) inhibitors [7]. Overexpression of human ABCG2 is shown to confer resistance against various, clinically-relevant compounds, including mitoxantrone, methotrexate, topotecan, irinotecan, imatinib, and flavopiridol [8]. This mechanisms has been associated with various types of leukemia and cancers of the breast, lung, and gastrointestinal system, among others [9,10]. MDCKII-BCRP cells with *ABCG2* overexpression were also found to be more vulnerable to exogenous oxidative stress induced by several oxidants. This finding can be important in the process of selective death induction within cells overexpressing *ABCG2* [11].

Beside detoxification, ABCG2 also plays other important transport roles. It regulates heme/porphyrins homeostasis by allowing the efflux of porphyrins from cells [12] and also the transport of endogenous folates, mostly mono-, di-, and tri-glutamates of folic acid [10]. In addition, ABCG2 is known as “urate transporter” because it regulates serum uric acid level by the excretion of urate [13].

Many studies suggest that the presence of certain polymorphic variants could alter the transport functions of ABCG2, thus influencing the pharmacokinetics and resistance associated with various chemotherapeutic agents [5,14]. In addition, a dysfunction of the transporter may also lead to the development of gout, a common disease characterized by sodium urate salt deposition, typically in or around the joints and the kidney, which can potentially lead to inflammatory arthritis, nephropathy and kidney stones. Such deposition of urate crystals occurs as a direct consequence of elevated urate serum levels, i.e., hyperuricemia, which itself also increases the risk of other diseases of the excretory and circulatory systems [15].

Furthermore, several *ABCG2* nonsense and frameshift mutations, identified in the last decade, are demonstrated to be responsible for a lack of the erythrocyte blood group antigen Jr^a^, which is a condition known in medicine for over 40 years. All of the null alleles introduce premature stop codons in the ATP-binding domain of ABCG2, resulting in the development of the Jr(a-) blood group phenotype [16,17]. Such individuals, i.e., those null for ABCG2, produce an ABCG2 alloantibody upon blood transfusion that can cause transfusion reactions, which can lead to fatal hemolytic diseases of the fetus and newborn in extreme cases [18].

The observed frequencies of *ABCG2* polymorphisms greatly differ between populations [19,20]. The most widely represented across different populations, and hence the most extensively studied due to their potential clinical relevance, have been the missense variants c.34G>A (p.Val12Met) and, especially, c.421C>A (p.Gln141Lys) [21]. Although the pharmacogenetic role of c.34G>A (p.Val12Met) single nucleotide polymorphisms (SNP) remains unclear, c.421C>A (p.Gln141Lys) has often been associated with altered protein function [22]. It appears to be very common in Asian populations, with reported allelic frequencies between 27% and 34%. Its frequency in Caucasian populations is approximately 10%, while is rare in sub-Saharan African and African-American populations, with frequencies of only 5% [23]. The genetic variation of the complete coding sequence has been most commonly described for Asian populations [24,25,26] and, among Caucasians, the Swedish [27] and Dutch populations have been fully studied [28].

The only *ABCG2* polymorphisms studied so far in the Polish population were the most common ones, *viz*. c.34G>A (p.Val12Met) and c.421C>A (p.Gln141Lys), described recently in two case-control studies of the same team, examining their association with peptic ulcers development [29] and multiple myeloma susceptibility [30]. However, their observed allelic frequencies, 0% of c.34G>A (p.Val12Met) and only 1% of c.421C>A (p.Gln141Lys), were significantly lower from those described previously in other Caucasian populations. In our opinion, this unexpected discrepancy should be clarified, whether actually frequencies of these SNPs in the Polish population significantly differ from other Caucasians, especially taking into account their clinical associations. Our preliminary analysis of the genetic variation of *ABCG2* in a Polish population revealed the occurrence of not only the most frequently-described polymorphisms but also a number of rare or clinically important variants. These premises all together prompted us to take a look more carefully at *ABCG2* genetic variation in the Polish population. Therefore, to present the first complete genetic profile of *ABCG2* in the Polish population we examine not only the common variants of this gene but all its coding fragments using High Resolution Melting (HRM). The findings describe virtually the almost entire coding sequence of *ABCG2* among 190 individuals representing the Polish population.

## 2. Material and Methods

### 2.1. Genomic DNA Samples

Human genomic DNA samples were derived from anonymous unrelated volunteers recruited during the years 2010–2012 from the whole of Poland as part of the TESTOPLEK research project. In 2013, the samples were registered in the BBMRI catalog as the POPULOUS collection (POPUlation—LOdz UniverSity Biobank) at Biobank Lab, Department of Molecular Biophysics, University of Lodz [31,32]. Each subject gave written informed consent to participate in the study and completed a questionnaire. The study was approved by the Research Bioethics Commission, University of Lodz (Decision no. 8/KBBN-UŁ/II/2014 and Statement of the Research Bioethics Commission, University of Lodz from 17 June 2010). All procedures were performed in accordance with the Declaration of Helsinki.

Saliva samples were collected from each individual into Oragene OG-500 DNA collection/storage receptacles (DNA Genotek, Kanata, ON, Canada). Genomic DNA was subsequently isolated using a MagNA Pure LC DNA Isolation Kit–Large Volume (Roche, Basel, Switzerland) and quantified using broad range Quant-iT™ dsDNA Broad Range Assay Kit (Invitrogen™, Carlsbad, CA, USA). All the DNA samples also underwent internal quality control by PCR for sex determination [33], which is a standard procedure in our laboratory. The final DNA concentration was normalized to 200 pg/µL. A group of 190 representative samples from the POPULOUS collection were randomly enrolled in the study and their DNA was collected on two 96-vial plates (each of plate also included single sample with molecular grade water as negative control).

### 2.2. ABCG2 Scanning Using HRM

*ABCG2* genetic variation was studied using HRM which is an efficient and sensitive method for scanning sequences [34]. The reaction was performed as was described previously [34], regarding mixture composition, conditions of PCR and melting, using 384-micro well plates and CFX384™ real-time PCR system (Bio-Rad Laboratories Inc., Hercules, CA, USA) in duplicate for each sample. The primers are presented in Table 1. The HRM melting curve analysis was performed in Bio-Rad Precision Melt Analysis Software, version 1.2 (Bio-Rad Laboratories Inc., Hercules, CA, USA). Melting results for each DNA plate have been analyzed separately to avoid false distinction [35]. To verify the obtained genetic variation results, several samples representing each melting cluster were selected for direct sequencing. In the event that fewer than four samples were obtained, all samples from cluster were used.

### 2.3. Verification of Scanning Results by DNA Sequencing

Preparation of samples for sequencing including reaction mixture and the conditions used for PCR, the samples purification and quality control procedures, have been described previously [34]. The list of primers used for direct sequencing is presented in Table 1. The samples were applied for sequencing reaction using BigDye Terminator V3.1 (Applied Biosystems, Foster City, CA, USA) according to the manufacturer’s protocol. The PCR-sequencing product was purified using BigDye X-Terminator kit following the manufacturer’s protocol (Applied Biosystems, Foster City, CA, USA). Then, 30 μL of each purified sample was applied to the 96 wells of the titration plates and analyzed using a 3500 Genetic Analyzer (Applied Biosystems, Foster City, CA, USA).

### 2.4. Detection of ABCG2 Polymorphisms

Sequencing results were analyzed by CodonCode Aligner software (CodonCode Corporation, Centerville, NY, USA) using *ABCG2* genomic DNA sequence NG_032067.2 obtained from GenBank (http://www.ncbi.nlm.nih.gov) as a reference sequence. Each of detected *ABCG2* polymorphisms were assigned the following parameters from GenBank, which were then used as nomenclature in present study: dbSNP IDs (rs numbers), the coding DNA nucleotide position based on the NM_004827.3 reference sequence, the amino acid position in the protein for SNPs in exons based on the NP_004818.2 reference sequence.

### 2.5. Inter-Population Comparison of Frequency of ABCG2 Polymorphisms

The population specific differences have been revealed and expressed as MAF values, confirmed by the chi-square test for alleles, with Yates’ correction if any allele quantity was below 5. The significance of the difference was shown as *p*-values, with values < 0.05 being regarded as significant. Comparison of obtained genetic variation results with literature data from ABCG2 scanning for other populations was performed for overlapped polymorphisms.

### 2.6. Predicting the Effect of Missense Variants

The effect of missense variants on protein functionality was predicted with common in silico tools: SIFT—which predicts whether an amino acid substitution is likely to affect protein function based on sequence homology and the physico-chemical similarity between alternate amino acids (https://sift.bii.a-star.edu.sg/); PolyPhen-2—which uses 5%/10% false positive rates for the HumDiv model and 10%/20% false positive rates for the HumVar model as the thresholds for classification (http://genetics.bwh.harvard.edu/pph2/dokuwiki/start); REVEL—an ensemble method for predicting the pathogenicity of missense variants which integrates scores from other tools (MutPred, FATHMM v2.3, VEST 3.0, PolyPhen-2, SIFT, PROVEAN, MutationAssessor, MutationTaster, LRT, GERP++, SiPhy, phyloP, and phastCons) (https://sites.google.com/site/revelgenomics/); MetaLR—an ensemble method that uses logistic regression to integrate the results of other components (SIFT, PolyPhen-2 HDIV, PolyPhen-2 HVAR, GERP++, MutationTaster, Mutation Assessor, FATHMM, LRT, SiPhy, PhyloP) (https://sites.google.com/site/jpopgen/dbNSFP).

### 2.7. Linkage Disequilibrium and Haplotype Blocks Analysis

The consistency of genotype distribution was verified for all the detected polymorphisms by the Hardy–Weinberg equilibrium (HWE) exact test assuming a *p*-value higher than 0.001. Linkage disequilibrium (LD) and haplotype block analysis was performed by Haploview 4.2 software (http://www.broad.mit.edu/mpg/haploview/). LD analysis was performed for each pair of polymorphisms using |D’| and r^2^ parameters. The obtained data for both these linkage parameters was compared with available population statistics using the LDmatrix Tool from the LDlink package (https://ldlink.nci.nih.gov/?tab=ldmatrix).

Haplotype block identification was performed based on the four-gamete test which indicates the occurrence of at least one historical recombination event and identify a set of contiguous and ordered SNP markers which demonstrate no evidence for recombination [36].

The consistency of the haplotype distribution was additionally verified by the MACH 1.0 tool (http://csg.sph.umich.edu/abecasis/MACH/index.html). Based on this data, network analysis was performed for haplotypes from each block with the Network 10 tool using the media-joining algorithm which was chosen as the most suitable approach (https://www.fluxus-engineering.com/sharenet.htm).

## 3. Results

### 3.1. Detected ABCG2 Genetic Variation

The *ABCG2* coding regions and exon/intron boundaries were scanned using HRM, as these were the most important gene areas. The maximal length of the melted PCR products was not longer than 250 bp to ensure the acceptable melting resolution suitable for polymorphisms detection. Therefore, several long exons were divided into separate fragments for analysis, giving a total of 24 areas successively scanned by HRM (Figures S1–S24 and Tables S1–S24). A total of 14 coding exons were fully scanned for each of the 190 subjects. Only the final coding fragment in exon 16 could not be scanned due to problems with the melting resolution caused by the presence of multiple melting domains and this was true even for very shortened length of PCR products. Likewise, exon 1 and the major part of exon 16 had to omitted due to them containing long, non-coding sequences, resulting in difficulties with PCR amplification caused by GC-rich composition among others.

**Figure 1:**
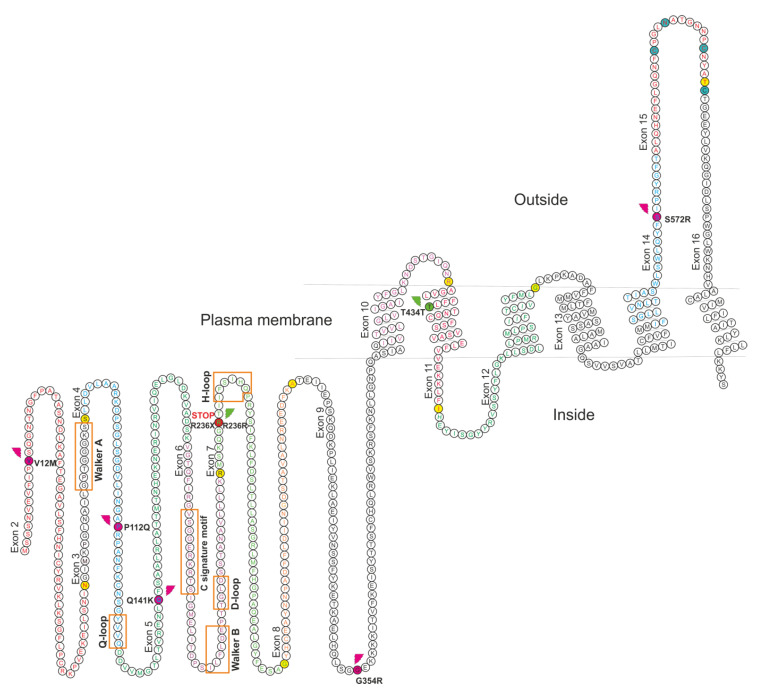
Structure of ABCG2 transporter in plasma membrane with highlighted amino acids for which genetic variants were detected in this study (based on the NP_004818.2 reference sequence). Amino acids encoded by subsequent exons are described and typed by different font color, and amino acids located on exons borders as splicing effect have background colored in yellow. Conserved motifs in Nucleotide Binding Domain: Walker A and B, the ABC signature C motif, Q-, D- and H-loops are described and indicated with orange squares. Amino acids involved in forming intra-molecular (C592-C608) and inter-molecular disulfide bonds (C603), and functional N-glycosylation site (N596) have background colored in blue. Amino acids with colored background and marks mean variants detected in this study: purple—missense, green—silent, red—nonsense mutation.

HRM scanning identified 17 variants. Two of these were located in the same nucleotide position but presented different molecular effects (c.706C>T, p.Arg236Ter, and c.706C>A, p.Arg236=) so they were counted in present study as separate SNPs (Table 2). Eight SNPs were found in the exons (Figure 1) and five of them are missense variants which change the amino acid sequence: c.34G>A (p.Val12Met), c.335C>A (p.Pro112Gln), c.421C>A (p.Gln141Lys), c.1060G>A (p.Gly354Arg), c.1714A>C (p.Ser572Arg). One SNP was also heterozygously detected as point-nonsense mutation resulting in a premature stop codon (c.706C>T, p.Arg236Ter) with the minor allele frequency (MAF) estimated as 0.003. Among the SNPs located in the exons, only one was found homozygously, c.421C>A (p.Gln141Lys) with MAF = 0.117. Eight SNPs were identified in the intron sequences, one of them homozygously c.1647+40T>C with MAF = 0.391. Moreover, one deletion variant was detected in an intron at c.690-19_690-17delTGT with MAF = 0.003.

The cytoplasmic nucleotide binding domain (NBD) of the protein, encompassing amino acids 1–395, was found to include four amino acids altered by missense variants and one premature stop variant: c.335C>A (p.Pro112Gln) located 25 amino acids downstream of the Walker A motif and 11 upstream of the Q-loop motif; variant c.421C>A (p.Gln141Lys), located 15 amino acids downstream of the Q-loop motif and 45 upstream of the C signature motif; SNP c.706C>T (p.Arg236Ter), located 25 amino acids downstream of Walker B motif, and three upstream of the H-loop motif. All of the motifs have been described as highly conserved and form the ATP catalytic center of the protein. SNP c.1714A> (p.Ser572Arg) was located in the extracellular domain, 20 amino acids upstream of the first cysteine which is crucial for forming intra-molecular disulfide bonds (Figure 1).

*In silico* analysis of all the missense variants detected in this study, incorporating several of the most common predictors, surprisingly classified three SNPs, *viz*. c.34G>A (p.Val12Met), c.421C>A (p.Gln141Lys) and c.1060G>A (p.Gly354Arg), as benign in protein functioning. However, two other variants, *viz*. c.335C>A (p.Pro112Gln) and c.1714A> (p.Ser572Arg), were assessed to be potentially damaging and having a possible deleterious effect on protein activity (Table S25).

### 3.2. Inter-Population Comparison of ABCG2 Genetic Variation

The studied Polish population demonstrated lower MAF values than those obtained in Asians, represented by Chinese [24], Japanese [26], and Korean populations [25], for the following SNPs: c.34G>A (p.Val12Met); c.203+36A>G; c.263+10A>G; c.421C>A (p.Gln141Lys); c.1367+20G>A (Table 3). Only SNP c.1647+40T>C did not significantly differ between the Polish population (MAF = 0.391) and the Japanese (MAF = 0.398, *p* = 0.840) and the Korean populations (MAF = 0.326, *p* = 0.135). It was nevertheless found to be significantly different to the Chinese population (MAF = 0.241, *p* = 0.033), however, this was probably only an effect of the small sample size included in that particular study. The Polish population demonstrated a higher MAF for c.532−16A>G (0.035); however, this SNP was only detected in the Japanese population (MAF = 0.003, *p* = 0.002). The comparison of a similar set of overlapping SNPs, *viz*. c.34G>A (p.Val12Met), c.203+36A>G, c.421C>A (p.Gln141Lys), c.1367+20G>A and c.1647+40T>C, did not reveal any significant differences between two Caucasian populations: the Polish and the Dutch [28] (Table 3).

These observations are consistent with those of studies from other populations, which were focused only on the most common variants c.34G>A (p.Val12Met) and c.421C>A (p.Gln141Lys). The Polish population demonstrated significantly lower MAFs for these SNPs (0.032 and 0.117, respectively) than in Asian populations, such as those from like Vietnam (MAF = 0.360 and MAF = 0.310, respectively) and another studied group from China (MAF = 0.330 and MAF = 0.290, respectively) [25]. However, the values were not significantly different to those from other Caucasian populations, such as those in Sweden (MAF = 0.020 and MAF = 0.100, respectively) [27], Hungary (MAF = 0.040, and MAF = 0.094, respectively) [37], the Czech Republic (MAF = 0.015, and MAF = 0.075, respectively) [38] and Germany (MAF = 0.030, [39] and MAF = 0.104 [40], respectively) (Table 3).

These observations also correspond with comparison of ethnic groups in general, available only for c.421C>A (p.Gln141Lys). The MAF among the Polish population (0.117) was found to be significantly lower than that for the Han Chinese population (MAF = 0.342, *p* <0.001), although its frequency was not significantly different than that observed for Caucasian Americans (MAF = 0.119, *p* = 0.938) and Europeans (MAF = 0.107, *p* = 0.738) and it was higher than African Americans (MAF = 0.053, *p* = 0.015) or Africans from the sub-Sahara region (MAF = 0.009, *p* < 0.001) [20] (Table 3).

### 3.3. Linkage Disequilibrium Analysis

**Figure 2:**
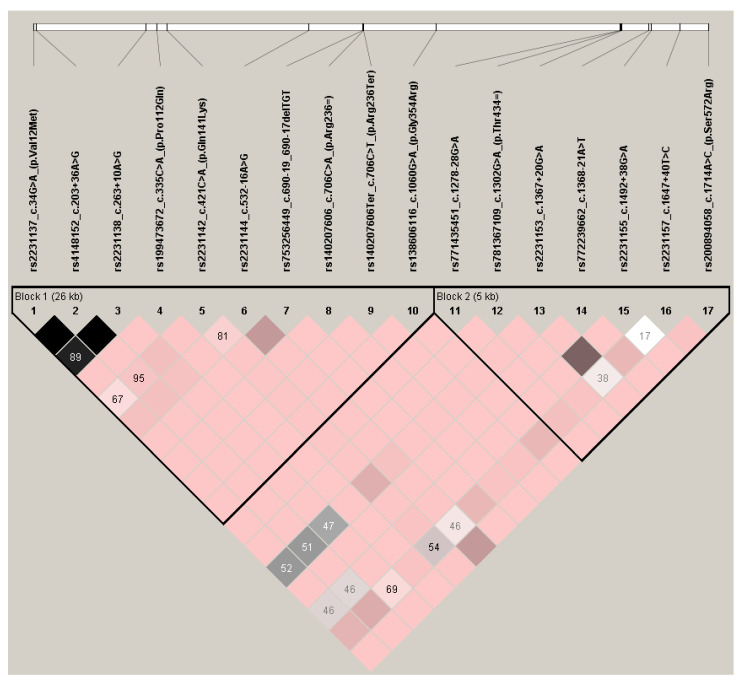
Linkage disequilibrium analysis of *ABCG2* polymorphisms detected in this study using |D’| parameter. Pairwise linkage demonstrated as |D’| values (100×) by graded pink and black colors, denser color means closer linkage and lack of value means |D’| = 1 × 100. Bolded triangles show haplotype blocks.

Linkage disequilibrium analysis was performed using r^2^ and |D’| statistics based on consistency with the HWE exact test for all detected variants. For the |D’| parameter, perfect linkage (|D’| = 1) was observed for the great majority of polymorphism pairs (122 from 136), while strong linkage (1 > |D’| ≥ 0.8) was detected for the next three pairs. Weak or absent linkage, as indicated by low |D’| values (|D’| <  0,8) was observed mostly between pairs of polymorphisms from c.34G>A (p.Val12Met), c.203+36A>G, c.263+10A>G, c.421C>A (p.Gln141Lys), c.532-16A>G and those from c.1367+20G>A, c.1492+38G>A, c.1647+40T>C (Figure 2).

**Figure 3:**
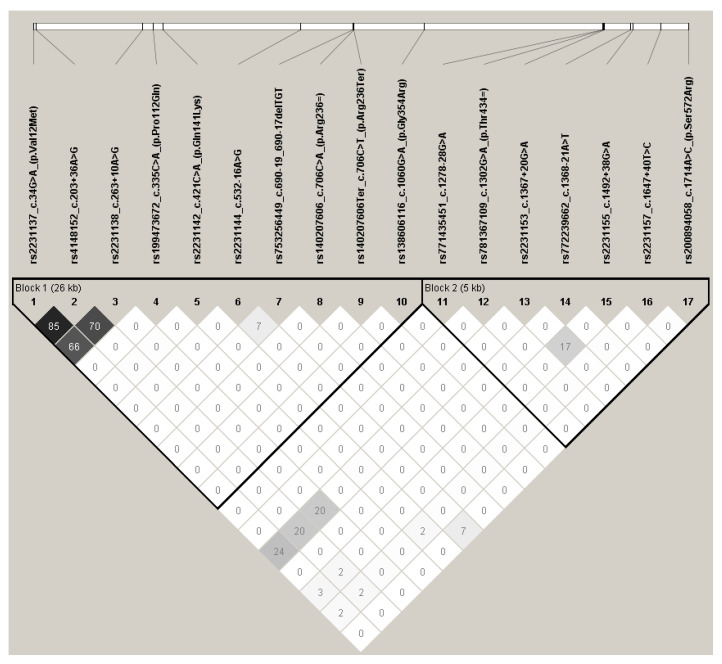
Linkage disequilibrium analysis of *ABCG2* polymorphisms detected in this study using r^2^ parameter. Pairwise linkage demonstrated as r^2^ values (×100) by graded black colors, denser color means closer linkage. Bolded triangles show haplotype blocks.

Regarding r^2^ parameter, no examples of perfect linkage (r^2^ = 1 were detected between any polymorphism pair. However, strong linkage was revealed between three SNPs: c.34G>A (p.Val12Met) and c.203+36A>G (r^2^  =  0.852); c.203+36A>G and c.263+10A>G (r^2^  =  0.706); c.34G>A (p.Val12Met) and c.263+10A>G (r^2^  =  0.665) (Figure 3).

These observations are in line with the linkage statistics obtained by LDlink for the 1000 Genomes Project database. Most polymorphism pairs demonstrated maximal |D’| values, with weaker values identified for the same pairs given above, regardless of the chosen 1000 Genomes population. Likewise, only SNP c.34G>A (p.Val12Met), c.203+36A>G and c.263+10A>G were strongly linked in terms of r^2^ parameter among Europeans (Figure S25a) and world population in general (Figure S25b).

### 3.4. Haplotype Block Estimation and Haplotype Analysis

The analysis of haplotype blocks revealed at least one recombination spot, which was clearly indicated upstream of SNP c.1367+20G>A (scanned exon 11). However, two other SNPs were detected closely upstream of the estimated recombination spot and because these were observed only in single samples so were arbitrarily included to the second haplotype block. Hence, two haplotype blocks were defined, the first one including detected polymorphisms from exon 2 to 9, and the second one, polymorphisms from exon 11 to 14. A total of seven haplotypes were identified whose frequencies were estimated as at least 1% (Table 4).

Block 1, spanning 26 kb, included 10 polymorphisms and four common haplotypes were established for them. The most frequent haplotype comprised only the major alleles of all polymorphisms (frequency: 0.804), followed by haplotype with minor allele A for SNP c.421C>A (p.Gln141Lys) (frequency: 0.113). The frequencies of the next two haplotypes were much lower, i.e., 0.031 for c.532-16A>G and 0.024 for c.203+36A>G (both, with minor alleles G). Block 2 encompassed seven SNPs over a distance of around 5 kb. The most frequent haplotype consisted of only major alleles of all SNPs (frequency: 0.584), followed by one with the minor allele C for SNP c.1647+40T>C (frequency: 0.376) and another with the minor allele G for c.1367+20G>A (frequency: 0.020). These five SNPs used as the basis for distinguishing the major haplotypes, were tagged as haplotype tag SNPs (htSNPs). A complete set of haplotypes and their frequencies are presented in Table S26.

The most common haplotype from block 1 and the two most frequent from block 2 demonstrated greater than 10% crossing. Other possible crossings between haplotypes with frequencies higher than 1% were observed between the second most common haplotype of block 1 and the two most common from block 2, between the third most common haplotype of block 1 and the most common from block 2, and between the fourth most common haplotype of block 1 and the second and third from block 2. The frequency of recombination between blocks was estimated as 0.40, calculated as the multiallelic D’ coefficient.

Network analysis found the most common haplotype in block 1 to act as core for the others. However, three closely-linked variants, *viz*. c.34G>A (p.Val12Met), c.203+36A>G and c.263+10A>G, demonstrated separate paths of divergence (Figure S26a). In block 2, the most common haplotype was found to act as the root for the two next most common but together, they all determine the origin for the remaining minor haplotypes (Figure S26b).

### 3.5. Inter-Population Comparison of ABCG2 Haplotypes

The adjusted haplotype profiles of Japanese [26] and Dutch populations [28] were also compared with those of the present study. Differences in the frequencies of the common haplotypes G-A-A-C, G-A-A-A, A-G-A-C and A-G-G-C for the overlapping SNPs in block 1, *viz*. c.34G>A (p.Val12Met), c.203+36A>G, c.263+10A>G, c.421C>A (p.Gln141Lys), were observed between the Polish (frequencies of 0.848, 0.115, 0.008, and 0.024, respectively) and Japanese populations (frequencies of 0.489, 0.319, 0.130, and 0.062, respectively). In contrast, haplotypes: G-A-C, G-A-A, A-G-C for the overlapping SNPs c.34G>A (p.Val12Met), c.203+36A>G, and c.421C>A (p.Gln141Lys) in block 1, were very similar between the Polish (0.848, 0.115 and 0.032) and Dutch populations (0.816, 0.119, and 0.065). Smaller differences were observed for common haplotype frequencies from block 2: haplotypes A-T, A-C, and G-T for the overlapping SNPs c.1367+20G>A and c.1647+40T>C, were 0.585, 0.386, and 0.024 in the present study and 0.441, 0.398, and 0.161 for the Japanese population and 0.493, 0.463, and 0.045 for the Dutch population.

## 4. Discussion

Almost the entire coding sequence of the *ABCG2* gene, together with its neighboring intronic fragments, was scanned using HRM in samples taken randomly from a population of 190 unrelated Polish individuals. The results identified the presence of 17 different polymorphisms within the gene, including rare ones and some of which have possible clinical significance.

Our results unambiguously demonstrate that the frequencies of the most common polymorphisms of *ABCG2* in the Polish population, which is exclusively Caucasian, do not differ significantly to other Caucasian populations and are lower than in Asian populations. This conclusion contradicts previous, and the only available so far results of *ABCG2* genetic variation in the Polish population, obtained by Niebudek, et al. [30] and Salagacka-Kubiak, et al. [29] for SNPs c.34G>A (p.Val12Met) and c.421C>A (p.Gln141Lys), with both studies probably using the same control group (*n* = 97). The obtained MAF values for the SNPs were 0 and 0.010, respectively, which were significantly lower than those established in our study: 0.032 (*p* = 0.027) and 0.117 (*p* < 0.001), respectively. Despite their results appearing surprisingly low compared to the current state of knowledge, the authors did not comment on these differences at all. It is possible that these inconsistencies could be attributed to sample size or bias effects such as too small study group or insufficiently randomized sample selection. However, very possible explanation of such underestimation would be also not enough sensitive genotyping method. Authors performed genotyping by method of restriction fragment length polymorphism (RFLP) which is still commonly used in laboratory practice, but does not include any additional step verifying results accuracy. Greater sensitivity can be achieved by methods based on qPCR such as HRM, used in the present study, which examine genetic variation in throughout the entire tested sequence, not only at selected points. In our previous study, HRM has also been demonstrated to be an efficient and sensitive method for scanning and genotyping polymorphic variants. We have successfully scanned the coding sequence of one of the most important ABC genes, *ABCC1*, for representatives of the Polish population, with the results being confirmed by targeted genotyping of selected SNPs [34]. Moreover, both HRM strategies ensure greater reliability, by including an additional control step, such as verification by direct sequencing. Although HRM also has some limitations, which we also described previously [35], which prevented us from scanning the final *ABCG2* coding part in exon 16, our findings demonstrate it to be an efficient technique to study *ABCG2* genetic variation. This conclusion is also supported by other research groups, which have successively used HRM for association studies of *ABCG2* polymorphisms with gout [41] and Jr(a-) phenotype [42]. Therefore, we determine the occurrence frequencies of SNPs c.34G>A (p.Val12Met) and c.421C>A (p.Gln141Lys) in the Polish population, which have been clearly defined in this study.

Due to their potential clinical relevance, the common *ABCG2* SNPs, c.34G>A (p.Val12Met) and c.421C>A (p.Gln141Lys), are the most extensively studied in vitro and in vivo research. Although the significance of c.34G>A (p.Val12Met) remains unclear, c.421C>A (p.Gln141Lys) has often been reported to be associated with altered protein functionality. Generally, lower levels of mRNA maturation, protein folding and membrane trafficking were observed compared to the wild-type protein expression in the cellular membrane [43] but this also occurred as a result of ubiquitination-mediated proteasomal degradation, which the protein undergoes when it is recognized as misfolded [44]. This processes probably occur as an effect of an amino acid change located within the functionally important ATP-binding region between the Walker A and B motifs of the protein which likely affects its ATPase activity levels [6]. Reduced ABCG2 expression as consequence of the presence of SNP c.421C>A (p.Gln141Lys) was associated with an adverse response to cancer chemotherapy, but its pharmacogenomic effect appears to be dependent on substrate and correlated with its genotype [6,15] which has been also well summarized recently elsewhere [23,44]. Among 67 Japanese patients with chronic phase of chronic myeloid leukemia (CML) with genotype CA or AA the dose-adjusted imatinib mesylate (IM) trough concentration was significantly higher than in those with genotype CC [45]. Authors suggested reduction of IM dose from 400 mg for patients with the wild-type CC genotype to 300 mg for genotype CA or AA to obtain a plasma threshold of approximately 1000 ng/mL [46]. In case of 67 Caucasian patients with solid malignancies and gastrointestinal stromal tumors, a 22% reduction in plasma clearance (imatinib and CGP74588) was observed in heterozygous CA (genotype AA not found) compared with wild-type CC patients [47]. In another study for 349 Caucasian patients with primary lung cancer (161 small cell lung cancer (SCLC), 187 nonsmall cell lung cancer (NSCLC) and 1 mixed), significantly worse overall survival was observed for carriers with A allele treated with platinum-based drugs [39]. In Kurdish case-control study in which 100 breast cancer patients and 200 healthy controls were enrolled, A allele were significantly more frequent in patients and those with AA genotype had higher risk of progressing breast cancer. In addition, patient with A allele had complete response to chemotherapeutic agents (treatments with anthracyclines and Paclitaxol) [48]. Furthermore, in Chinese study of 1169 breast cancer patients (anthracycline-based chemotherapy), genotype GA/AA of variant c.34G>A (p.Val12Met), genotype AA of variant c.421C>A (p.Gln141Lys) and their haplotypes A-C and G-A were significantly associated with increased risk for developing breast carcinoma [49]. The conclusion seems to be common for all of mentioned studies, that clinical effects of c.421C>A (p.Gln141Lys) are significant and frequently observed, regardless of populations, drugs or cancers. Hence, c.421C>A (p.Gln141Lys) is considered to be one of the most important variant, both in terms of cancer chemotherapy response and also drug toxicity and resistance [6]. However, there were suggested ethnic differences in clinical response between East Asians and Caucasians [44], e.g., the TKIs sunitinib and erlotinib induced more frequent adverse events at normal doses in East Asians than Caucasians, resulting in higher rates of treatment failure [50]. Unfortunately, neither clinical study has been performed yet for the Polish population, however, abovementioned examples clearly suggest, that targeted genotyping together with treatment response monitoring could be very important in choosing the best strategy of therapy and should be implemented in clinical approach.

Furthermore, a number of studies of different ethnic populations, including genome-wide association (GWA) studies, have also indicated c.421C>A (p.Gln141Lys) to be the major risk factor for gout and hyperuricemia, which is correlated with increased urate concentration [13,51,52,53]. Regarding SNP c.34G>A (p.Val12Met), although it did not alter the protein expression and urate transport activity [43], some high-throughput studies [41,54], supported by meta-analysis results [38], suggest that it plays a protective role against gout. Thus, determining the appropriate frequency of both of these *ABCG2* variants in each particular population appears to be an important step in determining the full clinical implications of the gene.

Moreover, the scan also found rare SNPs, including several missense ones. The in silico analysis based on common predictors suggested that two of them, c.335C>A (p.Pro112Gln) and c.1714A>C (p.Ser572Arg), may be deleterious, while interestingly, the most common *ABCG2* SNPs were indicated to be likely benign for protein functioning, in spite their abovementioned clinical associations. A recent functional study, examining SNP c.1714A>C (p.Ser572Arg), confirmed this prediction and reported that when present, the cells did not produce a matured glycoprotein and demonstrated decreased levels of ABCG2 protein in the plasma membrane [55]. This could result from the fact that the SNP is located near to conserved cysteine responsible for forming intra-molecular disulfide bound. Although similar effect could also be observed for variant c.335C>A (p.Pro112Gln) located near to important conserved motifs in NBD, to the best of our knowledge, does not appear to have been the subject of similar study. This may suggest, that some rare missense variants could have also clinical relevance and require functional studies to determine their assumed impact. Although such SNPs may have significant consequences for their carriers, their true influence in effective therapy is difficult to estimate as they observed frequency in population is quite low. However, for this reason, impact of such rare polymorphisms for the entire Polish population seems to be rather a little. Another missense variant c.1060G>A (p.Gly354Arg) was also found in the Japanese population with the same frequency (*n* = 177, MAF = 0.003, *p* = 0.508 − insignificant) [26] as in our study; however, it has indicated as being likely benign and has yet to be subjected to a functional study.

Furthermore, our present findings also indicate the presence of a heterozygous nonsense mutation c.706C>T (p.Arg236Ter) (MAF = 0.003) which was associated with the Jr(a-) blood group phenotype. So far, c.376C>T (p.Gln126Ter, rs72552713) nonsense mutation was reported the most frequently but is rather typical for Japanese population. Among Caucasians, the nonsense mutations c.706C>T (p.Arg236Ter), observed in the present study, c.730C>T (p.Gln244Ter, lack dbSNP ID) and c.736C>T (p.Arg246Ter, rs200190472) are more commonly identified [56]. Unfortunately, it was not possible to collect the medical data of the donors and hence to verify the sample phenotype. Nevertheless, this finding may suggest that variants involved with the Jr(a-) phenotype in the Polish population are no such phenomenon as we previously thought and may should be considered more seriously in transfusion.

To gain a fuller picture of the *ABCG2* in Polish population, linkage disequilibrium (LD) and haplotype block analysis were performed. The LD results indicated the presence of very similar dependencies to those observed for Europeans and general worldwide population, suggesting that its inheritance pattern is rather evolutionary conserved, regardless of population type. This conclusion is consistent with haplotype block analysis which defined the two studied blocks, the first spanning from exon 2 to exon 9 and the second from exon 11 to exon 14 to be retained across populations. These also contained the same cores: between exon 2 and 5 and between exon 11 and 15, with a recombination spot between them. The polymorphisms located between exon 5 and 11 have been variously classified, probably due to their rare inherence in this sequence fragment: they were classified in the first block, between the 5′ flanking region and exon 9, as in the present study and in the Japanese population [26]; however, they were found to be classified in the second block, between exon 5 and 15, in the Chinese study [24] and they were not classified in either block in the Korean study [25].

Population specific differences have been also observed in common haplotypes comparison. Their frequencies in the Polish population looked very similar as in Dutch population [28], whereas they greatly differed compared to Japanese population [26], especially in haplotype block 1. These findings support the conclusion that differences between populations do not concern single or common polymorphisms of *ABCG2*, but the great, inherited parts of the gene. For that reason, linkage disequilibrium and haplotype analysis can reveal potentially significant population-specific differences which can be used in identification of SNPs in routine screening to obtain precision treatment based on *ABCG2* polymorphisms genotype or haplotype [44].

## 5. Conclusions

In conclusion, in the present study almost entire *ABCG2* coding sequence was scanned using an efficient HRM method for 190 representatives of the Polish population. The findings indicate that the frequencies of most common SNPs in Polish populations did not differ significantly from those in other Caucasians, thus providing a clarification this issue of the distribution of the clinically-relevant SNPs c.34G>A (p.Val12Met) and c.421C>A (p.Gln141Lys). Additionally, a comprehensive analysis was performed of the genetic variation of *ABCG2* in the Polish population. Clinical implementation of *ABCG2* genotyping is crucial for the development of individualized therapy and the determination of accurate dosing guidelines in future. We believe that our findings may be helpful for other researchers evaluating *ABCG2* clinical associations and be of value for clinicians working to improve diagnostic or therapeutic efficacy.

## Figures and Tables

**Table 1:** Pairs of primers used for High Resolution Melting (HRM) screening and sequencing of *ABCG2*.

Exon	Number of Scanned bp Upstream from the Exon (without Primer)	Forward Primer (5′→3′)	Reverse primer (5′→3′)	Number of Scanned bp Downstream from the Exon (without Primer)	Size (bp) Including Primers	Polymorphism Detected in HRM Scanning
HRM scanning						
1	-	-	-	-	-	-
2	9 (28*)	TGGACTATCAACTTACTATTGCTTTTC	CAGCTCCTTCAGTAAATGCC		161	c.34G>A (rs2231137)
		GACAGCTTCCAATGACCTGA	TGGAAATAGCCAAAACCTGTG	54	213	c.203+36A>G (rs4148152)
3	23	GAACCTGACTTAAAATGGAATAGTAAA	AAAAGTGCACAGAAAACGCT	23	153	c.263+10A>G (rs2231138)
4	16	TCTTATAGGTTATTAGACCCACAACAT	GAAACAGAAAATGCAAACCCA	11	190	c.335C>A (rs199473672)
5	32	CATGGTCTTAGAAAAGACTCATTATCA	TGACCCTGTTAATCCGTTCG		174	c.421C>A (rs2231142)
		GAAAACTTACAGTTCTCAGCAGCTC	CCACACAGGGAAAGTCCTACT	37	178	-
6	22	GATAATGACTGGTTGTTATCATTGAC	GCTCCATTCCTATACTAGTCCTTTTT		115	c.532-16A>G (rs2231144)
		TTTATCCGTGGTGTGTCTGG	CCCCAAGAATATCTGGGACA	49	215	-
7	51	GGCAAGAATAGAGTATTTTACTGAGAA	TATCGAGGCTGATGAATGGA		129	c.706C>T/C>A (rs140207606) c.690-19_690-17delTGT (rs753256449)
		GCAGGGACGAACAATCATCT	AGGCCCGTGGAACATAAGTC		109	-
		TGATAGCCTCACCTTATTGGC	CTATTAATGAAGCATTTTACAGCATAA	15	125	-
8	32	TCTTTGTTTTCCAAGACCATCA	AGGCAAAAATCTGGGACTGT	15	191	-
9	22	TGTTGTTATCTTGTTTGTGTTTCC	GGAGTTGACATAAATCTCCGC		120	-
		CCAAGCAGGATAAGCCACTC	GCTGATCTCCTTGAAGACTGTG		140	c.1060G>A (rs138606116)
		AATTACATCAACTTTCCGGGG	AACCACATTGTTCCCATTTGA	26	199	-
10	24	TCTCTAATTGAAACTCTTCCCCTTT	TGAAGAAAGTAACAGCATTTTCTGA	24	181	-
11	60	TGTGGAAAGAGTTTTGTGGGTA	CTAACCAATAGCCCCTGCTG	37	229	c.1278-28G>A (rs771435451) c.1302G>A (rs781367109) c.1367+20G>A (rs2231153)
12	37	TGCCCTGTGGCTTCTTAAAC	CTTGGTAACATCCTCATGGGT		147	c.1368-21A>T (rs772239662)
		TCAGCGGATACTACAGAGTGTCA	CTGACTTCACCCATAGGCAA	40	174	c.1492+38G>A (rs2231155)
13	26	GTGACCTAGGCAGTTGGGTT	AACCACACTCTGACCTGCTG		156	-
		TGTTTACCCTTATGATGGTGGC	TCAGAGCAAACACAGTTCAGAC		145	-
		AGCAGGTCAGAGTGTGGTTT	TGTGCAGGAAGAAATGAAGGA	42	127	c.1647+40T>C (rs2231157)
14	10	GCAGGCCTGACTTTTAGTATTTG	TGTTCCTAGCTTGGGAATGC	29	172	c.1714A>C (rs200894058)
15	15	CTGTTTACCTTGCCCTGCTC	CAAAACCCATTTTGACACTGAA	11	151	-
16	-	-	-	-	-	-
Direct sequencing						
1		-	-		-	
2		GTTGTGCCTGTCTTCCCATT	TGACTTCATGATCTGCCTGC		719	
3		ATGCATGACCTGTTTTGTTTGTT	AAAGCAAGACACCATTGGCT		650	
4		AGCCAATGGTGTCTTGCTTT	ACCAGCAGACATTTCCAAACT		716	
5		AGTTTGGAAATGTCTGCTGGT	ACGTACAACACCACATTGCC		649	
6		AGTACAGTATGTGGGCCGGAAT	CCCCTACACCCTCATCACAG		718	
7		TGCAGATTACCTGGGTTGCT	CACATGCATGCACATTGAAA		721	
8		CGTGGGAAGAAGAGAGAAAGAA	GCAAATGCATATATTGCCAAG		535	
9		GGCCTGTTATACTTTTCTAATGACAGA	CTGAGTTCATGCCACCACAC		967	
10		TTTTCCAGCAGTGTGCTTTG	AGGCAGGAGAATTGCTTGAA		575	
11		AGGGCCCATCTTCAAATACC	TTGCTTGCTCTCTCCAACATT		732	
12		GAGAGGGAGGATGTGTGGAA	GGTTGAGGCTGCAGTGAGAT		727	
13		TTCCTGTGAAAAGAGAAGGCA	TCAAGAGAATCTGCCGATCA		797	
14		CAGTGCTTCTTGACCTTCCA	TGAACTGAAATGCAACAATGTG		709	
15		ATTGGGGAGAGAAGGAGGAA	CAGGAACCATAAGCCCTTGA		731	
16		-	-		-	

28*—upstream from the START codon. Due to observation of multiple melting domains for coding fragment of exon 16 in HRM scanning, despite several attempts, resulting no possibility to successively study its genetic variation, primers sequences for this fragments have been omitted.

**Table 2:** Summary of detected *ATP-binding cassette sub-family G member 2* (*ABCG2*) genetic variation using HRM.

Exon Scanned by HRM	dbSNP ID	Variant Position NM_004827.3:	Intron/Amino Acid Residue NP_004818.2:	Observed Genotypes ^a,b^ (*n*)			HWE Exact Test *p*-Value ^c^	MAF ^d^
				R/R	R/V	V/V		
2	rs2231137	c.34G>A	p.Val12Met	175	12	0	1	(A) 0.032
2	rs4148152	c.203+36A>G	Intron	175	14	0	1	(G) 0.037
3	rs2231138	c.263+10A>G	Intron	177	10	0	1	(G) 0.027
4	rs199473672	c.335C>A	p.Pro112Gln	184	1	0	1	(A) 0.003
5	rs2231142	c.421C>A	p.Gln141Lys	147	38	3	0.954	(A) 0.117
6	rs2231144	c.532-16A>G	Intron	174	13	0	1	(G) 0.035
7	rs140207606	c.706C>T	p.Arg236Ter	186	1	0	1	(T) 0.003
7	rs140207606	c.706C>A	p.Arg236=	186	1	0	1	(A) 0.003
7	rs753256449	c.690-19_690-17delTGT	Intron	186	1	0	1	(delTGT) 0.003
9	rs138606116	c.1060G>A	p.Gly354Arg	187	1	0	1	(A) 0.003
11	rs771435451	c.1278-28G>A	Intron	187	1	0	1	(A) 0.003
11	rs781367109	c.1302G>A	p.Thr434=	187	1	0	1	(A) 0.003
11	rs2231153	c.1367+20G>A	Intron	0	11	177	1	(G) 0.029
12	rs772239662	c.1368-21A>T	Intron	188	1	0	1	(T) 0.003
12	rs2231155	c.1492+38G>A	Intron	187	2	0	1	(A) 0.005
13	rs2231157	c.1647+40T>C	Intron	68	93	27	0.734	(C) 0.391
14	rs200894058	c.1714A>C	p.Ser572Arg	186	1	0	1	(C) 0.003

^a^ Number of genotypes detected during this study, R—reference allele, V—variant allele. ^b^ Total number of examined samples was 190, however during scanning single samples were excluded due to accidental problems with amplification during PCR and hence, total number of genotypes does not equal precisely 190. ^c^ *p*-value is consistent with Hardy–Weinberg equilibrium if *p* > 0.001. ^d^ Minor allele shown in brackets with its frequency.

**Table 3:** Inter-population comparison of minor allele frequency (MAF) values of *ABCG2* polymorphisms.

Population	*n*	rs2231137 c.34G>A (p.Val12Met)	rs4148152 c.203+36A>G	rs2231138 c.263+10A>G	rs2231142 c.421C>A (p.Gln141Lys)	rs2231144 c.532-16A>G	rs138606116 c.1060G>A (p.Gly354Arg)	rs2231153 c.1367+20G>A	rs2231157 c.1647+40T>C
Polish (current study)	190	0.032	0.037	0.027	0.117	0.035	0.003	0.029	0.391
Chinese [24]	27	0.204 (*p* < 0.001)	0.259 (*p* < 0.001)	0.204 (*p* < 0.001)	0.222 (*p* = 0.032)	-	-	0.315 (*p* < 0.001)	**0.241** **(*p* = 0.033)**
Japanese [26]	177	0.192 (*p* < 0.001)	0.192 (*p* < 0.001)	0.068 (*p* = 0.009)	0.319 (*p* < 0.001)	**0.003** **(*p* = 0.002)**	0.003 (*p* = 0.508)	0.161 (*p* < 0.001)	0.398 (*p* = 0.840)
Korean [25]		0.230 (*p* < 0.001, *n* = 275)	0.250 (*p* < 0.001, *n* = 92)	0.098 (*p* < 0.001, *n* = 92)	0.280 (*p* < 0.001, *n* = 275)	-	-	0.240 (*p* < 0.001, *n* = 92)	0.326 (*p* = 0.135, *n* = 92)
Dutch [28]	100	0.065 (*p* = 0.066)	0.065 (*p* = 0.130)	-	0.120 (*p* = 0.916)	-	-	0.045 (*p* = 0.326)	0.465 (*p* = 0.086)
Vietnamese [25]	140	0.360 (*p* < 0.001)	-	-	0.310 (*p* < 0.001)	-	-	-	-
Chinese [25]	191	0.330 (*p* < 0.001)	-	-	0.290 (*p* < 0.001)	-	-	-	-
Swedish [27]	60	0.020 (*p* = 0.376)	-	-	0.100 (*p* = 0.608)	-	-	-	-
Hungarian [37]	149	0.040 (*p* = 0.570)	-	-	0.094 (*p* = 0.336)	-	-	-	-
Czech [38]	100	0.015 (*p* = 0.221)	-	-	0.075 (*p* = 0.113)	-	-	-	-
German		0.030 (*p* = 0.862, *n* = 348) [39]	-	-	0.104 (*p* = 0.440, *n* = 1552) [40]	-	-	-	-
Han Chinese [20]	95	-	-	-	0.342 (*p* < 0.001)	-	-	-	-
American Caucasian [20]	88	-	-	-	0.119 (*p* = 0.938)	-	-	-	-
European Caucasian [20]	84	-	-	-	0.107 (*p* = 0.738)	-	-	-	-
African American [20]	94	-	-	-	**0.053 (*p* = 0.015)**	-	-	-	-
African (sub-Sahara) [20]	938	-	-	-	**0.009 (*p* < 0.001)**	-	-	-	-

Shaded cells mean significantly higher MAF than in the Polish population; bolded values mean significantly lower MAF than in the Polish population.

**Table 4:** Haplotype block analysis.

	Block 1												Block 2							
Variant residue ^a,b^	c.34G>A p.Val12Met	c.203+36A>G	c.263+10A>G	c.335C>A p.Pro112Gln	c.421C>A p.Gln141Lys	c.532-16A>G	c.706C>T p.Arg236Ter	c.706C>A p.Arg236=	c.690-19_690-17delTGT ^e^	c.1060G>A p.Gly354Arg	Haplotype frequency	Recombination between blocks ^d^	c.1278-28G>A	c.1302G>A p.Thr434=	c.1367+20G>A	c.1368-21A>T	c.1492+38G>A	c.1647+40T>C	c.1714A>C p.Ser572Arg	Haplotype frequency
Haplotypes ^c^	G	A	A	C	C	A	C	C	TGT	G	0.804	0.40	G	G	A	A	G	T	A	0.584
	G	A	A	C	A	A	C	C	TGT	G	0.113		G	G	A	A	G	C	A	0.376
	G	A	A	C	C	G	C	C	TGT	G	0.031		G	G	G	A	G	T	A	0.020
	A	G	G	C	C	A	C	C	TGT	G	0.024		others							0.020
	others										0.028									

^a^ Residues followed by reference sequences for coding nucleotide NM_004827.3 and amino acid position NP_004818.2. ^b^ Bolded polymorphisms mean haplotype tag SNPs (htSNPs). ^c^ Major alleles in white boxes, minor alleles in shaded boxes; all the haplotypes with their frequencies in population; haplotypes below 1% not shown and grouped like “others”. ^d^ Level of recombination between blocks as a value of multiallelic D’. ^e^ TGT allele in haplotype means no deletion.

## Supplementary Materials

The following are available online at https://www.mdpi.com/2073-4425/11/10/1144/s1, Figures S1–S24: HRM plots of *ABCG2* scanning for each scanned area, Figure S25: Linkage disequilibrium statistics r2 and |D’| from LDlink for 1000 Genomes Project database, for Europeans (a) and general world population (b), Figure S26: Network analysis of *ABCG2* haplotypes detected in this study for block 1 (a) and block 2 (b), Tables S1–S24: Summary of *ABCG2* scanning for each scanned area, Table S25: Nonsynonymous variants detected in this study and their predicted effect on protein functioning classified by in silico tools, Table S26: All the observed haplotypes in this study and their frequencies in *ABCG2* blocks.
