# Supplementary material for: Comprehensive Analysis of ABCG2 Genetic Variation in the Polish Population and Its Inter-Population Comparison
Source: Genes (Basel). 2020 Sep 29;11(10):1144. doi: 10.3390/genes11101144 (PMC7600124; doi:10.3390/genes11101144)
Supplement: Supplementary file 1 [file genes-11-01144-s001.zip › FigS1_S24_and_TabS1_S24_HRM_summary.pdf]

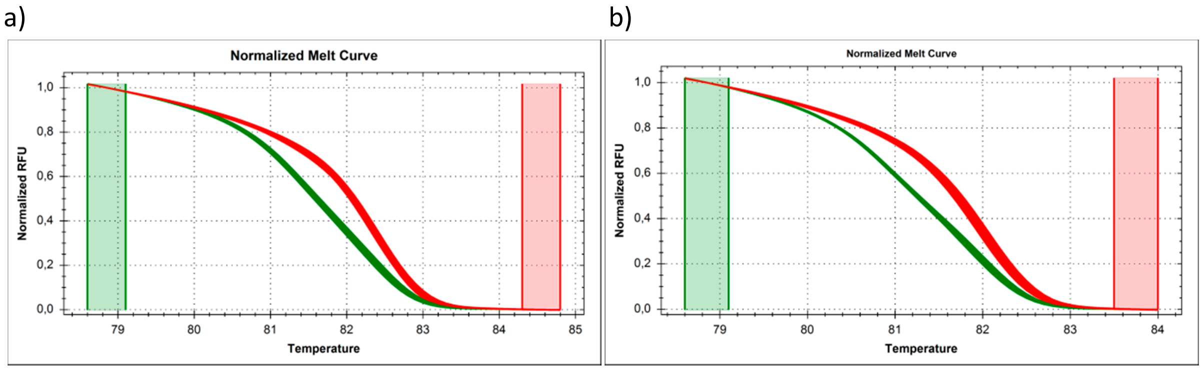


**Figure S1 Summary of *ABCG2* exon 2 scanning by HRM: a), b) melting plots for the first scanned area of the exon (NC_000004.11: 89061202 - 89061042, including primers).**

**Table S1 Summary of *ABCG2* exon 2 (the first area) scanning by HRM. Variant position according to reference sequence NM_004827.3. Scan rate is ratio between positive clustered and verified samples to all the scanned samples.**

| | c.34G>A | Plate 1 | Plate 2 | Overall |
|---|---|---|---|---|
| Samples scanned | | 95 | 95 | 190 |
| Melting clusters | | 2 | 2 | 4 |
| Samples in cluster 1 (red) | GG | 86 | 89 | 175 |
| Samples in cluster 2 (green) | GA | 7 | 5 | 12 |
| Samples excluded | | 2 | 1 | 3 |
| Scan rate | | - | - | 0.9842 |

a) b)

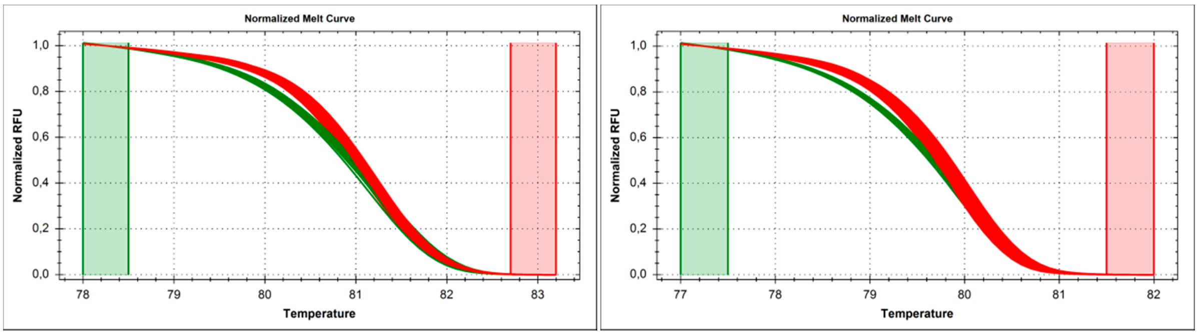


**Figure S2 Summary of *ABCG2* exon 2 scanning by HRM: a), b) melting plots for the second scanned area of the exon (NC_000004.11: 89061082 - 89060870, including primers).**

**Table S2 Summary of *ABCG2* exon 2 (the second area) scanning by HRM. Variant position according to reference sequence NM_004827.3. Scan rate is ratio between positive clustered and verified samples to all the scanned samples.**

| | c.203+36A>G | Plate 1 | Plate 2 | Overall |
|---|---|---|---|---|
| Samples scanned | | 95 | 95 | 190 |
| Melting clusters | | 2 | 2 | 4 |
| Samples in cluster 1 (red) | AA | 86 | 89 | 175 |
| Samples in cluster 2 (green) | AG | 9 | 5 | 14 |
| Samples excluded | | 0 | 1 | 1 |
| Scan rate | | - | - | 0.9947 |

a) 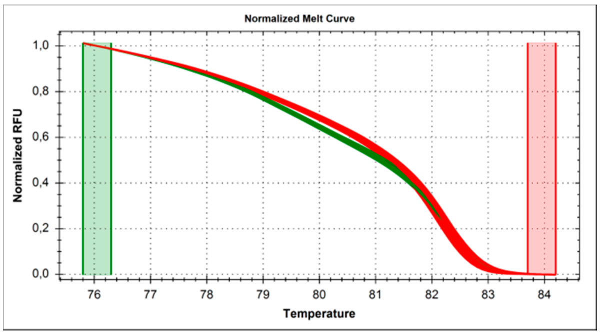


b) 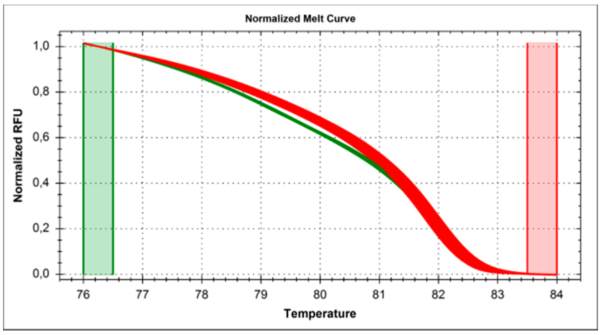


**Figure S3 Summary of *ABCG2* exon 3 scanning by HRM: a), b) melting plots for the whole exon scanned area (NC_000004.11: 89053837 - 89053685, including primers).**

**Table S3 Summary of *ABCG2* exon 3 scanning by HRM. Variant position according to reference sequence NM_004827.3. Scan rate is ratio between positive clustered and verified samples to all the scanned samples.**

| | c.263+10A>G | Plate 1 | Plate 2 | Overall |
|---|---|---|---|---|
| Samples scanned | | 95 | 95 | 190 |
| Melting clusters | | 2 | 2 | 4 |
| Samples in cluster 1 (red) | AA | 86 | 91 | 177 |
| Samples in cluster 2 (green) | AG | 7 | 3 | 10 |
| Samples excluded | | 2 | 1 | 3 |
| Scan rate | | - | - | 0.9842 |

a) b)

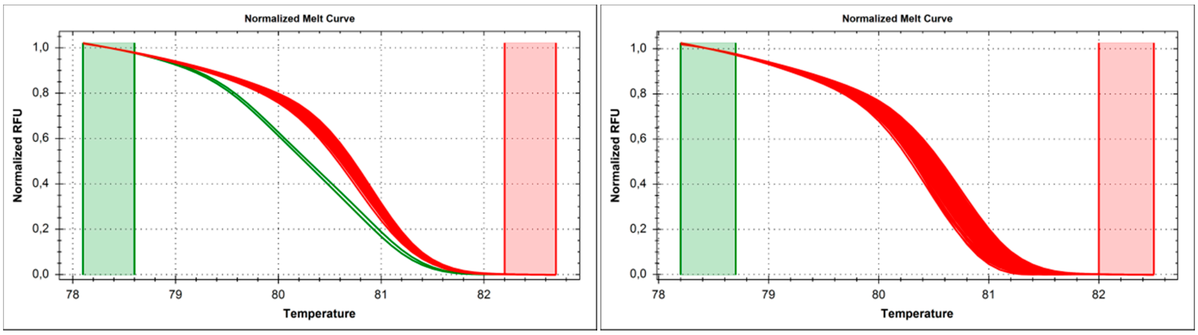


**Figure S4 Summary of *ABCG2* exon 4 scanning by HRM: a), b) melting plots for the whole exon scanned area (NC_000004.11: 89053112 - 89052923, including primers).**

**Table S4 Summary of *ABCG2* exon 4 scanning by HRM. Variant position according to reference sequence NM_004827.3. Scan rate is ratio between positive clustered and verified samples to all the scanned samples.**

| | c.335C>A | Plate 1 | Plate 2 | Overall |
|---|---|---|---|---|
| Samples scanned | | 95 | 95 | 190 |
| Melting clusters | | 2 | 1 | 3 |
| Samples in cluster 1 (red) | CC | 92 | 92 | 184 |
| Samples in cluster 2 (green) | CA | 1 | 0 | 1 |
| Samples excluded | | 2 | 3 | 5 |
| Scan rate | | - | - | 0.9737 |

a) b)

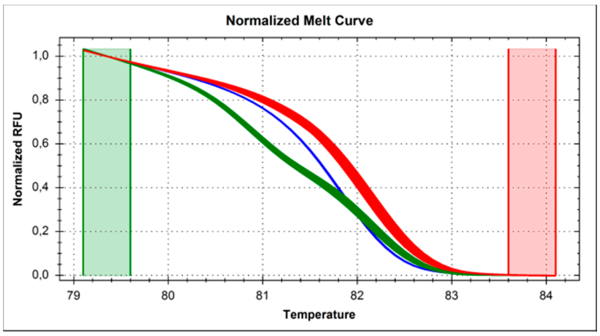


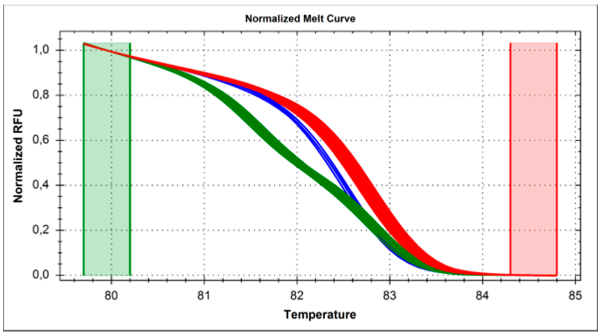


**Figure S5 Summary of *ABCG2* exon 5 scanning by HRM: a), b) melting plots for the first scanned area of the exon (NC_000004.11: 89052424 - 89052251, including primers)**

**Table S5 Summary of *ABCG2* exon 5 (the first area) scanning by HRM. Variant position according to reference sequence NM_004827.3. Scan rate is ratio between positive clustered and verified samples to all the scanned samples.**

| | c.421C>A | Plate 1 | Plate 2 | Overall |
|---|---|---|---|---|
| Samples scanned | | 95 | 95 | 190 |
| Melting clusters | | 3 | 3 | 6 |
| Samples in cluster 1 (red) | CC | 79 | 68 | 147 |
| Samples in cluster 2 (green) | CA | 15 | 23 | 38 |
| Samples in cluster 3 (blue) | AA | 1 | 2 | 3 |
| Samples excluded | | 0 | 2 | 2 |
| Scan rate | | - | - | 0.9895 |

a) b)

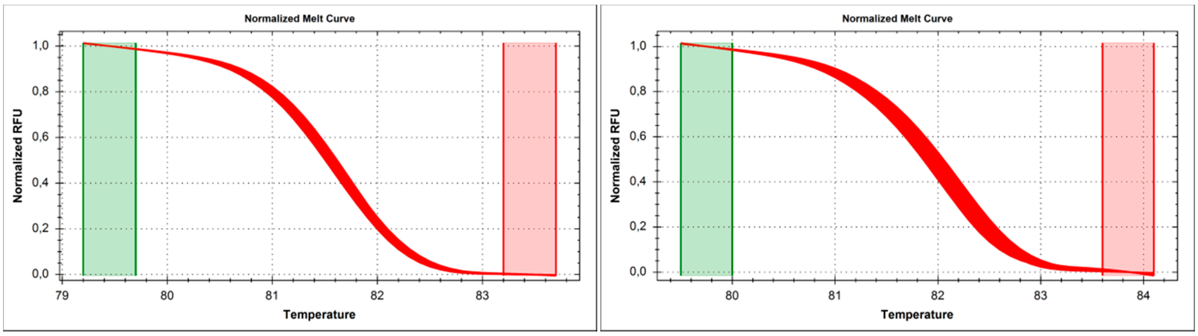


**Figure S6 Summary of *ABCG2* exon 5 scanning by HRM: a), b) melting plots for the second scanned area of the exon (NC_000004.11: 89052332 - 89052155, including primers)**

**Table S6 Summary of *ABCG2* exon 5 (the second area) scanning by HRM. No variant detected according to reference sequence NM_004827.3. Scan rate is ratio between positive clustered and verified samples to all the scanned samples.**

| | | Plate 1 | Plate 2 | Overall |
|---|---|---|---|---|
| Samples scanned | | 95 | 95 | 190 |
| Melting clusters | | 1 | 1 | 2 |
| Samples in cluster 1 (red) | wild type | 93 | 94 | 187 |
| Samples excluded | | 2 | 1 | 3 |
| Scan rate | | - | - | 0.9842 |

a) b)

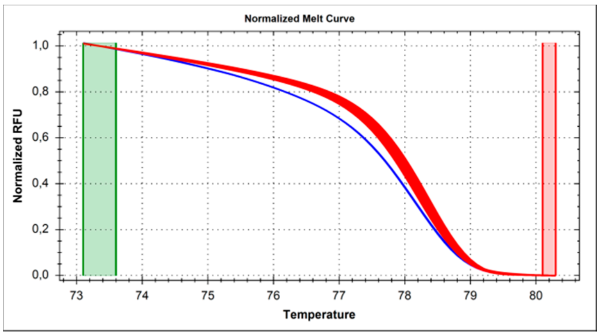


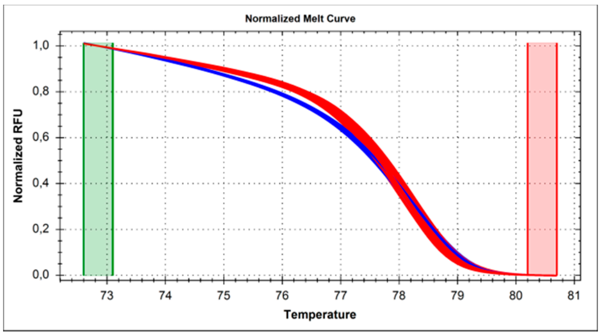


**Figure S7 Summary of *ABCG2* exon 6 scanning by HRM: a), b) melting for the first scanned area of the exon (NC_000004.11: 89042992 - 89042878, including primers).**

**Table S7 Summary of *ABCG2* exon 6 (the first area) scanning by HRM. Variant position according to reference sequence NM_004827.3. Scan rate is ratio between positive clustered and verified samples to all the scanned samples.**

| | c.532-16A>G | Plate 1 | Plate 2 | Overall |
|---|---|---|---|---|
| Samples scanned | | 95 | 95 | 190 |
| Melting clusters | | 2 | 2 | 4 |
| Samples in cluster 1 (red) | AA | 92 | 82 | 174 |
| Samples in cluster 2 (blue) | AG | 1 | 12 | 13 |
| Samples excluded | | 2 | 1 | 3 |
| Scan rate | | - | - | 0.9842 |

a) b)

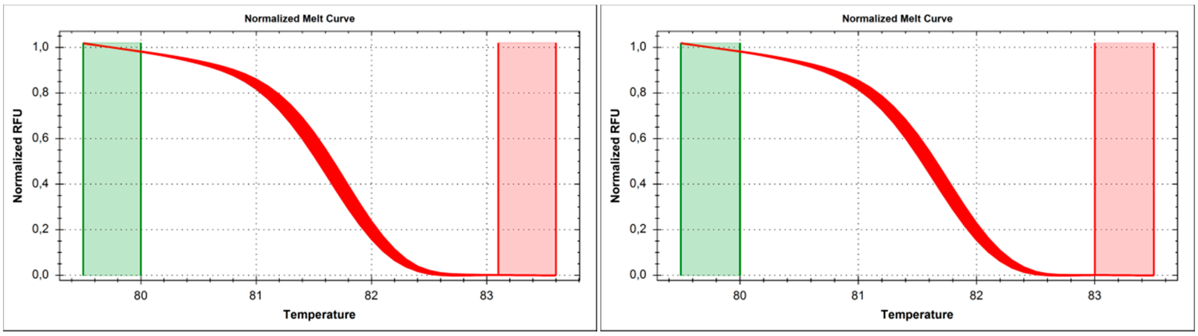


**Figure S8 Summary of *ABCG2* exon 6 scanning by HRM: a), b) melting for the second scanned area of the exon (NC_000004.11: 89042932 - 89042718, including primers).**

**Table S8 Summary of *ABCG2* exon 6 (the second area) scanning by HRM. No variant detected according to reference sequence NM_004827.3. Scan rate is ratio between positive clustered and verified samples to all the scanned samples.**

| | | Plate 1 | Plate 2 | Overall |
|---|---|---|---|---|
| Samples scanned | | 95 | 95 | 190 |
| Melting clusters | | 1 | 1 | 2 |
| Samples in cluster 1 (red) | wild type | 92 | 94 | 186 |
| Samples excluded | | 3 | 1 | 4 |
| Scan rate | | - | - | 0.9789 |

a)

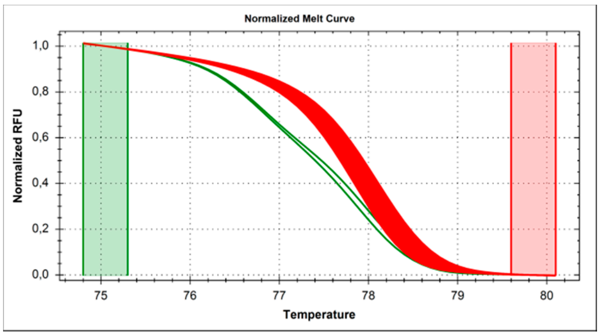


b)

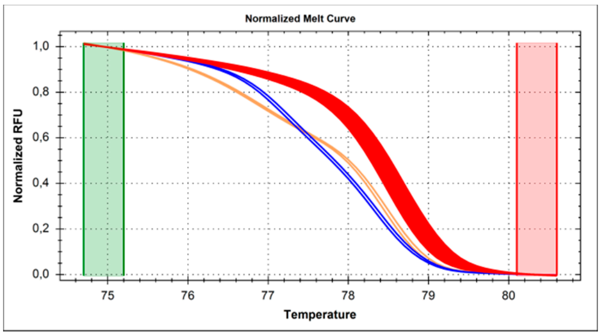


**Figure S9 Summary of *ABCG2* exon 7 scanning by HRM: a), b) melting plots for the first scanned area of the exon (NC_000004.11: 89039490 - 89039362, including primers).**

**Table S9 Summary of *ABCG2* exon 7 (the first area) scanning by HRM. Variants position according to reference sequence NM_004827.3. Scan rate is ratio between positive clustered and verified samples to all the scanned samples.**

| | c.706C>T c.706C>A | c.690-19_690-17delTGT | Plate 1 | Plate 2 | Overall |
|---|---|---|---|---|---|
| Samples scanned | | | 95 | 95 | 190 |
| Melting clusters | | | 2 | 3 | 5 |
| Samples in cluster 1 (red) | CC | TGT | 92 | 92 | 184 |
| Samples in cluster 2 (green) | CT | TGT | 1 | 0 | 1 |
| Samples in cluster 2 (blue) | CA | TGT | 0 | 1 | 1 |
| Samples in cluster 2 (orange) | CC | delTGT | 0 | 1 | 1 |
| Samples excluded | | | 2 | 1 | 3 |
| Scan rate | | | - | - | 0.9842 |

a) b)

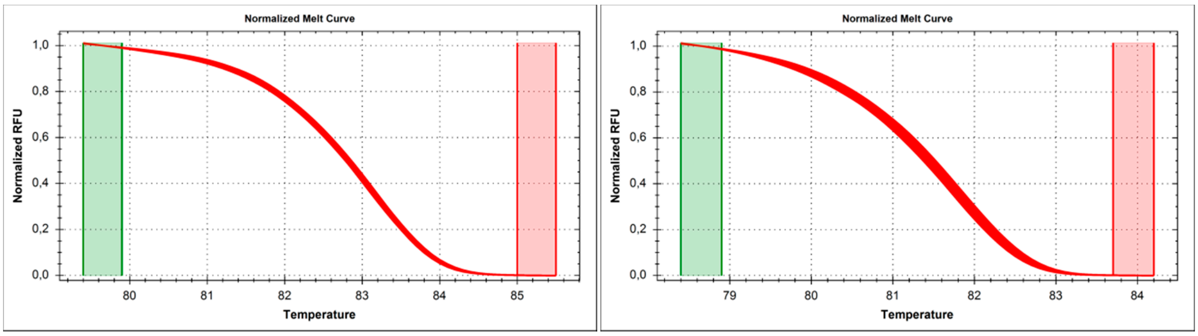


**Figure S10 Summary of *ABCG2* exon 7 scanning by HRM: a), b) melting plots for the second scanned area of the exon (NC_000004.11: 89039403 - 89039295, including primers).**

**Table S10 Summary of *ABCG2* exon 7 (the second area) scanning by HRM. No variant detected according to reference sequence NM_004827.3. Scan rate is ratio between positive clustered and verified samples to all the scanned samples.**

| | | Plate 1 | Plate 2 | Overall |
|---|---|---|---|---|
| Samples scanned | | 95 | 95 | 190 |
| Melting clusters | | 1 | 1 | 2 |
| Samples in cluster 1 (red) | wild type | 95 | 93 | 188 |
| Samples excluded | | 0 | 2 | 2 |
| Scan rate | | - | - | 0.9895 |

a) b)

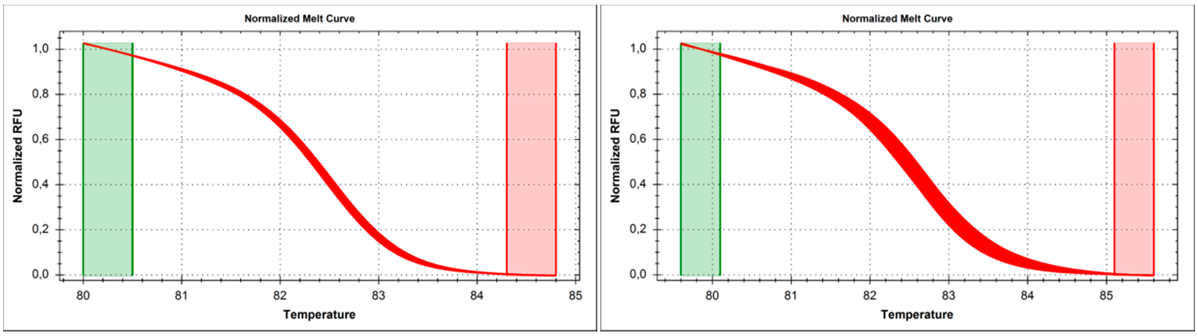


**Figure S11 Summary of *ABCG2* exon 7 scanning by HRM: a), b) melting plots for the third scanned area of the exon (NC_000004.11: 89039343 - 89039219, including primers).**

**Table S11 Summary of *ABCG2* exon 7 (the third area) scanning by HRM. No variant detected according to reference sequence NM_004827.3. Scan rate is ratio between positive clustered and verified samples to all the scanned samples.**

| | | Plate 1 | Plate 2 | Overall |
|---|---|---|---|---|
| Samples scanned | | 95 | 95 | 190 |
| Melting clusters | | 1 | 1 | 2 |
| Samples in cluster 1 (red) | wild type | 93 | 94 | 187 |
| Samples excluded | | 2 | 1 | 3 |
| Scan rate | | - | - | 0.9842 |

a) b)

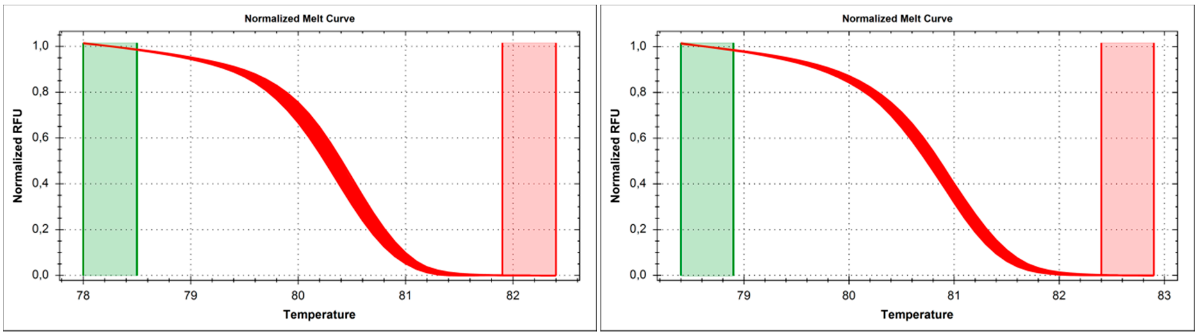


**Figure S12 Summary of *ABCG2* exon 8 scanning by HRM: a), b) melting plots for the whole exon scanned area (NC_000004.11: 89036264 - 89036074, including primers).**

**Table S12 Summary of *ABCG2* exon 8 scanning by HRM. No variant detected according to reference sequence NM_004827.3. Scan rate is ratio between positive clustered and verified samples to all the scanned samples.**

| | | Plate 1 | Plate 2 | Overall |
|---|---|---|---|---|
| Samples scanned | | 95 | 95 | 190 |
| Melting clusters | | 1 | 1 | 2 |
| Samples in cluster 1 (red) | wild type | 93 | 94 | 187 |
| Samples excluded | | 2 | 1 | 3 |
| Scan rate | | - | - | 0.9842 |

a) b)

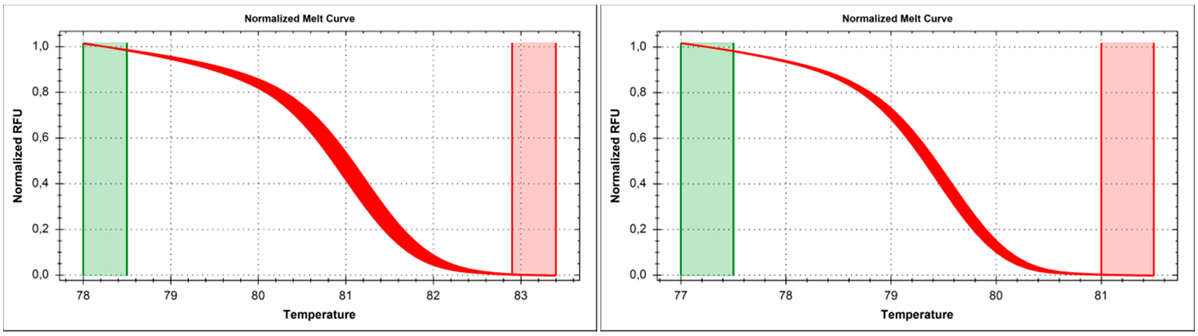


**Figure S13 Summary of *ABCG2* exon 9 scanning by HRM: a), b) melting plots for the first scanned area of the exon (NC_000004.11: 89034751 - 89034632, including primers).**

**Table S13 Summary of *ABCG2* exon 9 (the first area) scanning by HRM. No variant detected according to reference sequence NM_004827.3. Scan rate is ratio between positive clustered and verified samples to all the scanned samples.**

| | | Plate 1 | Plate 2 | Overall |
|---|---|---|---|---|
| Samples scanned | | 95 | 95 | 190 |
| Melting clusters | | 1 | 1 | 2 |
| Samples in cluster 1 (red) | wild type | 95 | 94 | 189 |
| Samples excluded | | 0 | 1 | 1 |
| Scan rate | | - | - | 0.9947 |

a) b)

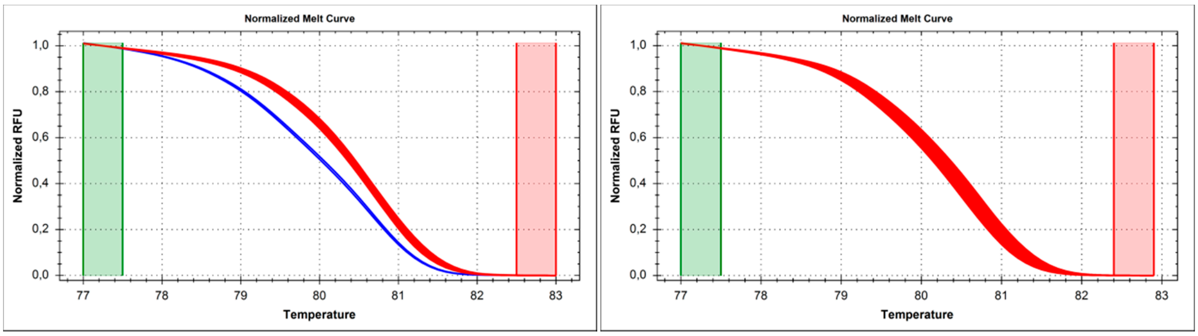


**Figure S14 Summary of *ABCG2* exon 9 scanning by HRM: a), b) melting for the second scanned area of the exon (NC_000004.11: 89034684 - 89034545, including primers).**

**Table S14 Summary of *ABCG2* exon 9 (the second area) scanning by HRM. Variant position according to reference sequence NM_004827.3. Scan rate is ratio between positive clustered and verified samples to all the scanned samples.**

| | c.1060G>A | Plate 1 | Plate 2 | Overall |
|---|---|---|---|---|
| Samples scanned | | 95 | 95 | 190 |
| Melting clusters | | 2 | 1 | 3 |
| Samples in cluster 1 (red) | GG | 94 | 93 | 187 |
| Samples in cluster 2 (blue) | GA | 1 | 0 | 1 |
| Samples excluded | | 0 | 2 | 2 |
| Scan rate | | - | - | 0.9895 |

a) b)

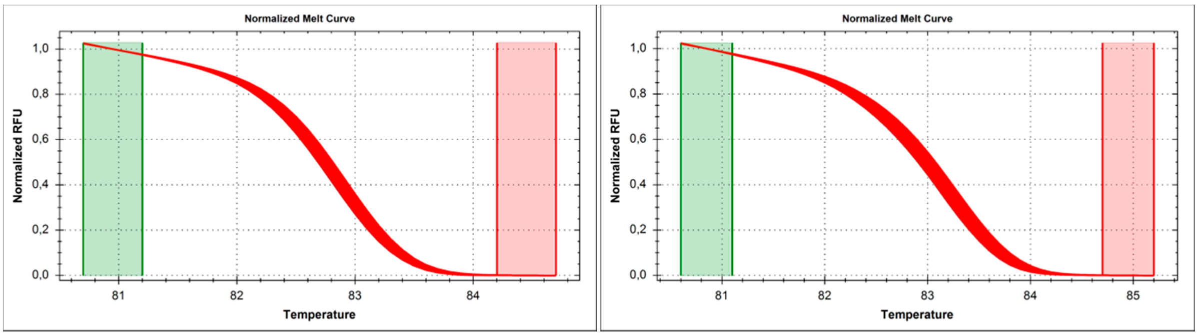


**Figure S15 Summary of *ABCG2* exon 9 scanning by HRM: a), b) melting plots for the third scanned area of the exon (NC_000004.11: 89034606 - 89034408, including primers).**

**Table S15 Summary of *ABCG2* exon 9 (the third area) scanning by HRM. No variant detected according to reference sequence NM_004827.3. Scan rate is ratio between positive clustered and verified samples to all the scanned samples.**

| | | Plate 1 | Plate 2 | Overall |
|---|---|---|---|---|
| Samples scanned | | 95 | 95 | 190 |
| Melting clusters | | 1 | 1 | 2 |
| Samples in cluster 1 (red) | wild type | 93 | 94 | 187 |
| Samples excluded | | 2 | 1 | 3 |
| Scan rate | | - | - | 0.9842 |

a) b)

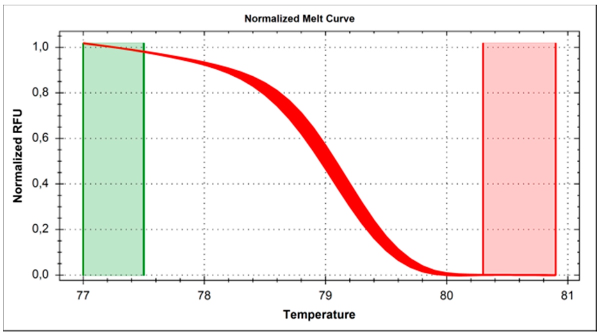


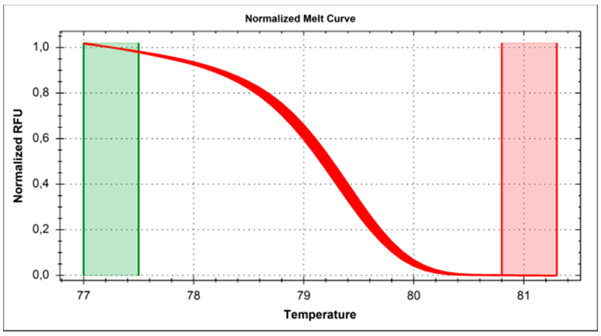


**Figure S16 Summary of *ABCG2* exon 10 scanning by HRM: a), b) melting plots for the whole exon scanned area (NC_000004.11: 89028467 - 89028287, including primers).**

**Table S16 Summary of *ABCG2* exon 10 scanning by HRM. No variant detected according to reference sequence NM_004827.3. Scan rate is ratio between positive clustered and verified samples to all the scanned samples.**

| | | Plate 1 | Plate 2 | Overall |
|---|---|---|---|---|
| Samples scanned | | 95 | 95 | 190 |
| Melting clusters | | 1 | 1 | 2 |
| Samples in cluster 1 (red) | wild type | 93 | 94 | 187 |
| Samples excluded | | 2 | 1 | 3 |
| Scan rate | | - | - | 0.9842 |

a) b)

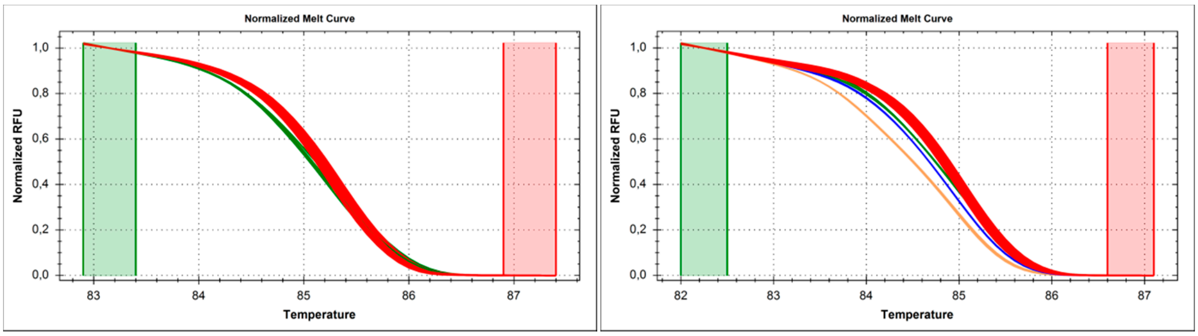


**Figure S17 Summary of *ABCG2* exon 11 scanning by HRM: a), b) melting plots for the whole exon scanned area (NC_000004.11: 89022553 - 89022325, including primers).**

**Table S17 Summary of *ABCG2* exon 11 scanning by HRM. Variants position according to reference sequence NM_004827.3. Scan rate is ratio between positive clustered and verified samples to all the scanned samples.**

| | c.1278-28G>A | c.1302G>A | c.1367+20G>A | Plate 1 | Plate 2 | Overall |
|---|---|---|---|---|---|---|
| Samples scanned | | | | 95 | 95 | 190 |
| Melting clusters | | | | 2 | 4 | 6 |
| Samples in cluster 1 (red) | GG | GG | AA | 90 | 85 | 175 |
| Samples in cluster 2 (green) | GG | GG | GA | 5 | 6 | 11 |
| Samples in cluster 2 (blue) | GA | GG | AA | 0 | 1 | 1 |
| Samples in cluster 2 (orange) | GG | GA | AA | 0 | 1 | 1 |
| Samples excluded | | | | 0 | 2 | 2 |
| Scan rate | | | | - | - | 0.9895 |

a) b)

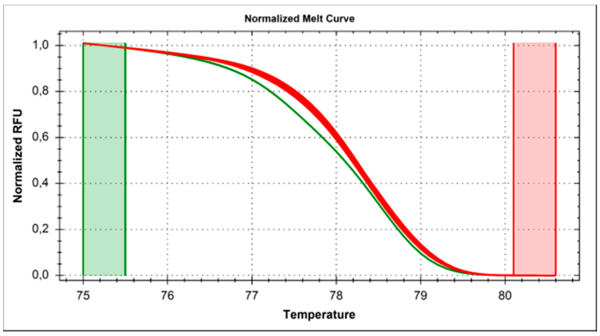


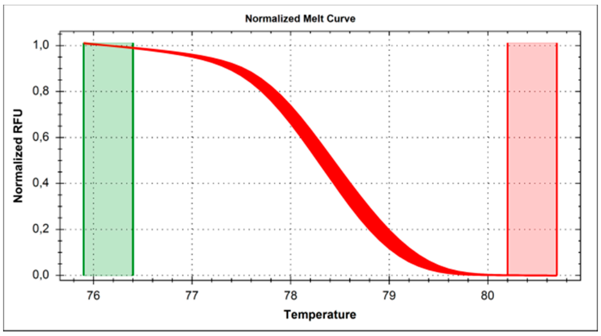


**Figure S18 Summary of *ABCG2* exon 12 scanning by HRM: a), b) melting plots for the first scanned area of the exon (NC_000004.11: 89020657 - 89020511, including primers).**

**Table S18 Summary of *ABCG2* exon 12 (the first area) scanning by HRM. Variant position according to reference sequence NM_004827.3. Scan rate is ratio between positive clustered and verified samples to all the scanned samples.**

| | c.1368-21A>T | Plate 1 | Plate 2 | Overall |
|---|---|---|---|---|
| Samples scanned | | 95 | 95 | 190 |
| Melting clusters | | 2 | 1 | 3 |
| Samples in cluster 1 (red) | AA | 94 | 94 | 188 |
| Samples in cluster 2 (green) | AT | 1 | 0 | 1 |
| Samples excluded | | 0 | 1 | 1 |
| Scan rate | | - | - | 0.9947 |

a) b)

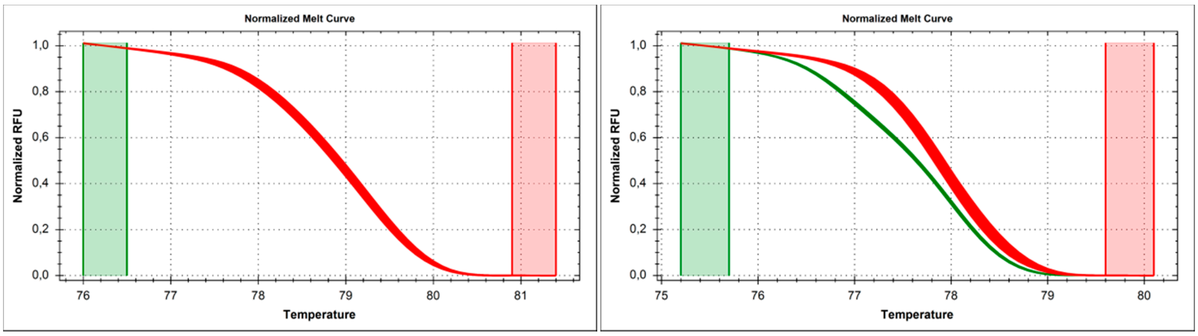


**Figure S19 Summary of *ABCG2* exon 12 scanning by HRM: a), b) melting plots for the second scanned area of the exon (NC_000004.11: 89020589 - 89020416, including primers).**

**Table S19 Summary of *ABCG2* exon 12 (the second area) scanning by HRM. Variant position according to reference sequence NM_004827.3. Scan rate is ratio between positive clustered and verified samples to all the scanned samples.**

| | c.1492+38G>A | Plate 1 | Plate 2 | Overall |
|---|---|---|---|---|
| Samples scanned | | 95 | 95 | 190 |
| Melting clusters | | 1 | 2 | 3 |
| Samples in cluster 1 (red) | GG | 95 | 92 | 187 |
| Samples in cluster 2 (green) | GA | 0 | 2 | 2 |
| Samples excluded | | 0 | 1 | 1 |
| Scan rate | | - | - | 0.9947 |

a) b)

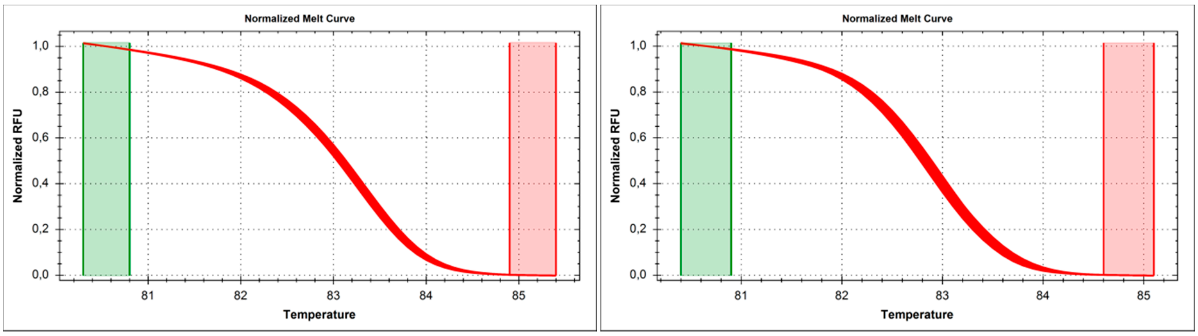


**Figure S20 Summary of *ABCG2* exon 13 scanning by HRM: a), b) melting plots for the first scanned area of the exon (NC_000004.11: 89018805 - 89018650, including primers).**

**Table S20 Summary of *ABCG2* exon 13 (the first area) scanning by HRM. No variant detected according to reference sequence NM_004827.3. Scan rate is ratio between positive clustered and verified samples to all the scanned samples.**

| | | Plate 1 | Plate 2 | Overall |
|---|---|---|---|---|
| Samples scanned | | 95 | 95 | 190 |
| Melting clusters | | 1 | 1 | 2 |
| Samples in cluster 1 (red) | wild type | 95 | 94 | 189 |
| Samples excluded | | 0 | 1 | 1 |
| Scan rate | | - | - | 0.9947 |

a) b)

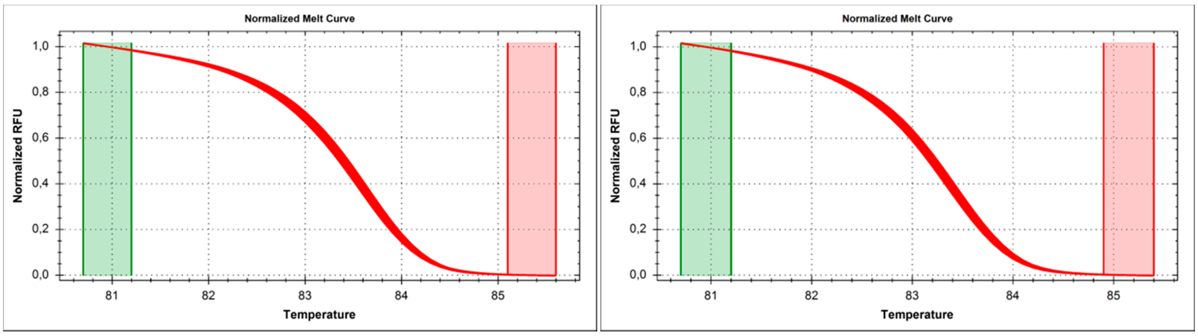


**Figure S21 Summary of *ABCG2* exon 13 scanning by HRM: a), b) melting plots for the second scanned area of the exon (NC_000004.11: 89018723 - 89018579, including primers).**

**Table S21 Summary of *ABCG2* exon 13 (the second area) scanning by HRM. No variant detected according to reference sequence NM_004827.3. Scan rate is ratio between positive clustered and verified samples to all the scanned samples.**

| | | Plate 1 | Plate 2 | Overall |
|---|---|---|---|---|
| Samples scanned | | 95 | 95 | 190 |
| Melting clusters | | 1 | 1 | 2 |
| Samples in cluster 1 (red) | wild type | 95 | 93 | 188 |
| Samples excluded | | 0 | 2 | 2 |
| Scan rate | | - | - | 0.9895 |

a) b)

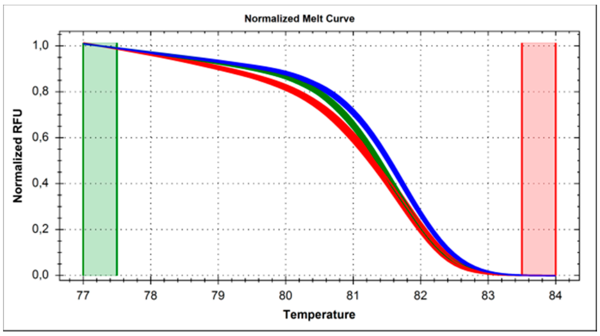


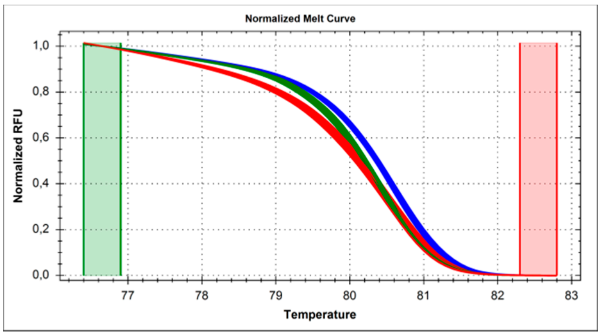


**Figure S22 Summary of *ABCG2* exon 13 scanning by HRM: a), b) melting plots for the third scanned area of the exon (NC_000004.11: 89018668 - 89018542, including primers)**

**Table S22 Summary of *ABCG2* exon 13 (the third area) scanning by HRM. Variant position according to reference sequence NM_004827.3. Scan rate is ratio between positive clustered and verified samples to all the scanned samples.**

| | c.1647+40T>C | Plate 1 | Plate 2 | Overall |
|---|---|---|---|---|
| Samples scanned | | 95 | 95 | 190 |
| Melting clusters | | 3 | 3 | 6 |
| Samples in cluster 1 (red) | TC | 48 | 45 | 93 |
| Samples in cluster 2 (green) | TT | 30 | 38 | 68 |
| Samples in cluster 3 (blue) | CC | 17 | 10 | 27 |
| Samples excluded | | 0 | 2 | 2 |
| Scan rate | | - | - | 0.9895 |

a)

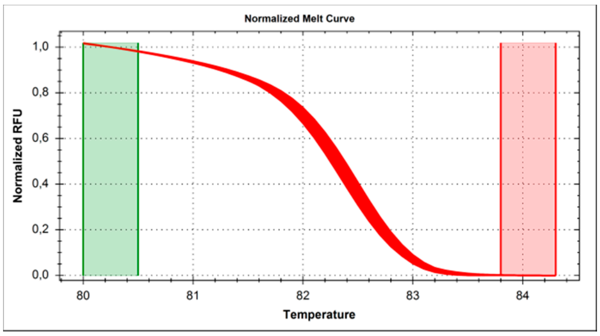


b)

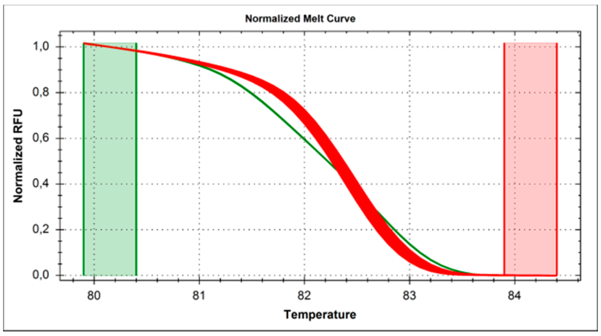


**Figure S23 Summary of *ABCG2* exon 14 scanning by HRM: a), b) melting plots for the whole exon scanned area (NC_000004.11: 89016794 - 89016623, including primers).**

**Table S23 Summary of *ABCG2* exon 14 scanning by HRM. Variant position according to reference sequence NM_004827.3. Scan rate is ratio between positive clustered and verified samples to all the scanned samples.**

| | c.1714A>C | Plate 1 | Plate 2 | Overall |
|---|---|---|---|---|
| Samples scanned | | 95 | 95 | 190 |
| Melting clusters | | 1 | 2 | 3 |
| Samples in cluster 1 (red) | AA | 93 | 93 | 186 |
| Samples in cluster 2 (green) | AC | 0 | 1 | 1 |
| Samples excluded | | 2 | 1 | 3 |
| Scan rate | | - | - | 0.9842 |

a) b)

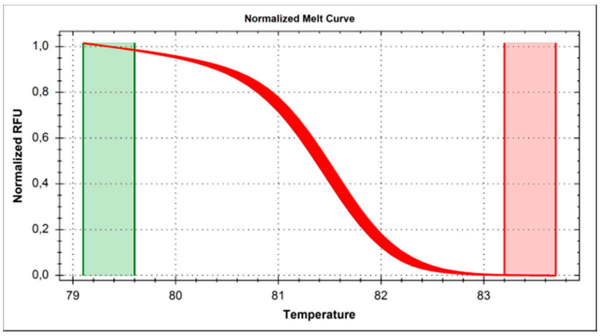


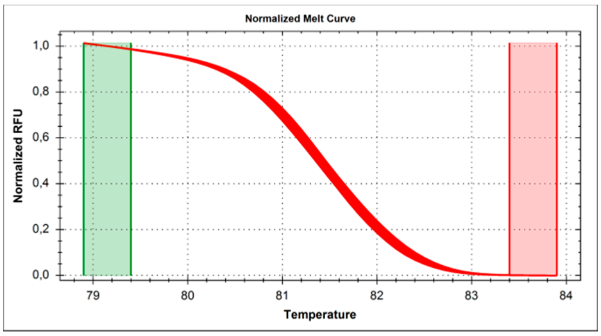


**Figure S24 Summary of *ABCG2* exon 15 scanning by HRM: a), b) melting plots for the whole exon scanned area (NC_000004.11: 89015846 - 89015696, including primers).**

**Table S24 Summary of *ABCG2* exon 15 scanning by HRM. No variant detected according to reference sequence NM_004827.3. Scan rate is ratio between positive clustered and verified samples to all the scanned samples.**

| | | Plate 1 | Plate 2 | Overall |
|---|---|---|---|---|
| Samples scanned | | 95 | 95 | 190 |
| Melting clusters | | 1 | 1 | 2 |
| Samples in cluster 1 (red) | wild type | 93 | 89 | 182 |
| Samples excluded | | 2 | 6 | 8 |
| Scan rate | | - | - | 0.9579 |
