# Supplementary material for: Comprehensive Analysis of ABCG2 Genetic Variation in the Polish Population and Its Inter-Population Comparison
Source: Genes (Basel). 2020 Sep 29;11(10):1144. doi: 10.3390/genes11101144 (PMC7600124; doi:10.3390/genes11101144)
Supplement: Supplementary file 1 [file genes-11-01144-s001.zip › FigS25_and_S26_descriptions.pdf]

Figure S25. Linkage disequilibrium statistics r2 and |D'| from LDlink for 1000 Genomes Project database, for Europeans (a) and average world population (b).

Due to absence in database some rare variants, including detected in this study: c.690-19_690-17delTGT, c.1278-28G>A, c.1302G>A (p.Thr434=), c.1368-21A>T, c.1714A>C (p.Ser572Arg), they were omitted in this comparison.

Figure S26. Network analysis of *ABCG2* haplotypes detected in this study for block 1 (a) and block 2 (b).

Each circle represents particular haplotype and its area corresponds with defined frequency. Haplotypes description base on numeration from Table S26. Variants determining subsequent haplotypes marked in each connection, htSNPs are bolded.
