# Supplementary material for: Comprehensive Analysis of ABCG2 Genetic Variation in the Polish Population and Its Inter-Population Comparison
Source: Genes (Basel). 2020 Sep 29;11(10):1144. doi: 10.3390/genes11101144 (PMC7600124; doi:10.3390/genes11101144)
Supplement: Supplementary file 1 [file genes-11-01144-s001.zip › TabS25_S26.pdf]

Table S25 Nonsynonymous variants detected in this study and their predicted effect on protein functioning classified by *in silico* tools

	SIFT		PolyPhen-2 HumDiv		PolyPhen-2 HumVar		REVEL		MetaLR	
c.34G>A (p.Val12Met)	1	tolerated	0	benign	0.001	benign	0.180	likely benign	0	tolerated
c.335C>A (p.Pro112Gln)	0	deleterious	0.995	probably damaging	0.978	probably damaging	0.425	likely benign (suspected)	0.774	damaging
c.421C>A (p.Gln141Lys)	0.19	tolerated	0.372	benign	0.216	benign	0.106	likely benign	0	tolerated
c.1060G>A (p.Gly354Arg)	0.53	tolerated	0.016	benign	0.006	benign	0.088	likely benign	0.191	tolerated
c.1714A>C (p.Ser572Arg)	0	deleterious	0.997	probably damaging	0.989	probably damaging	0.897	likely disease causing	0.687	damaging

SIFT version 5.2.2: a score < 0.05 are called 'deleterious' and all others are called 'tolerated'

PolyPhen-2 v2.2.2r398 HumDiv train model: 0 - 0.452 (benign) < 0.453 – 0.956 (possibly damaging) < 0.957 - 1 (probably damaging)

PolyPhen-2 v2.2.2r398 HumVar train model: 0 - 0.446 (benign) < 0.447 – 0.908 (possibly damaging) < 0.909 - 1 (probably damaging)

REVEL - scores above 0.5 called as 'likely disease causing' and scores below 0.5 as 'likely benign' (recommended the actual score is used when assessing a variant and a cut-off appropriate to chosen requirements)

MetaLR - scores above 0.5 called as 'damaging' and scores below 0.5 as 'tolerated'

Table S26 All the observed haplotypes in this study and their frequencies in *ABCG2* blocks

Block 1			Block 2		
Name	Haplotype	Frequency	Name	Haplotype	Frequency
1.1	GAACCACC(TGT)G	0.804	2.1	GGAAGTA	0.584
1.2	GAACAACC(TGT)G	0.113	2.2	GGAAGCA	0.376
1.3	GAACCGCC(TGT)G	0.031	2.3	GGGAGTA	0.020
1.4	AGGCCACC(TGT)G	0.024	2.4	GGGAGCA	0.006
1.5	AGACCACC(TGT)G	0.009	2.5	GGATGCA	0.003
1.6	GAACCATC(TGT)G	0.003	2.6	AGAAGTA	0.003
1.7	GAAACACC(TGT)G	0.003	2.7	GGAAACA	0.003
1.8	GGACCACC(TGT)G	0.003	2.8	GGAAGTC	0.003
1.9	GGGCCACC(TGT)G	0.003	2.9	GGGAATA	0.003
1.10	GAACCACC(TGT)A	0.003	2.10	GAAAGCA	0.003
1.11	GAACCACA(TGT)G	0.003			
1.12	GAACCGCC(delTGT)G	0.003			
