# Supplementary figures and images for: Comprehensive Analysis of ABCG2 Genetic Variation in the Polish Population and Its Inter-Population Comparison
Source: Genes (Basel). 2020 Sep 29;11(10):1144. doi: 10.3390/genes11101144 (PMC7600124; doi:10.3390/genes11101144)

a)

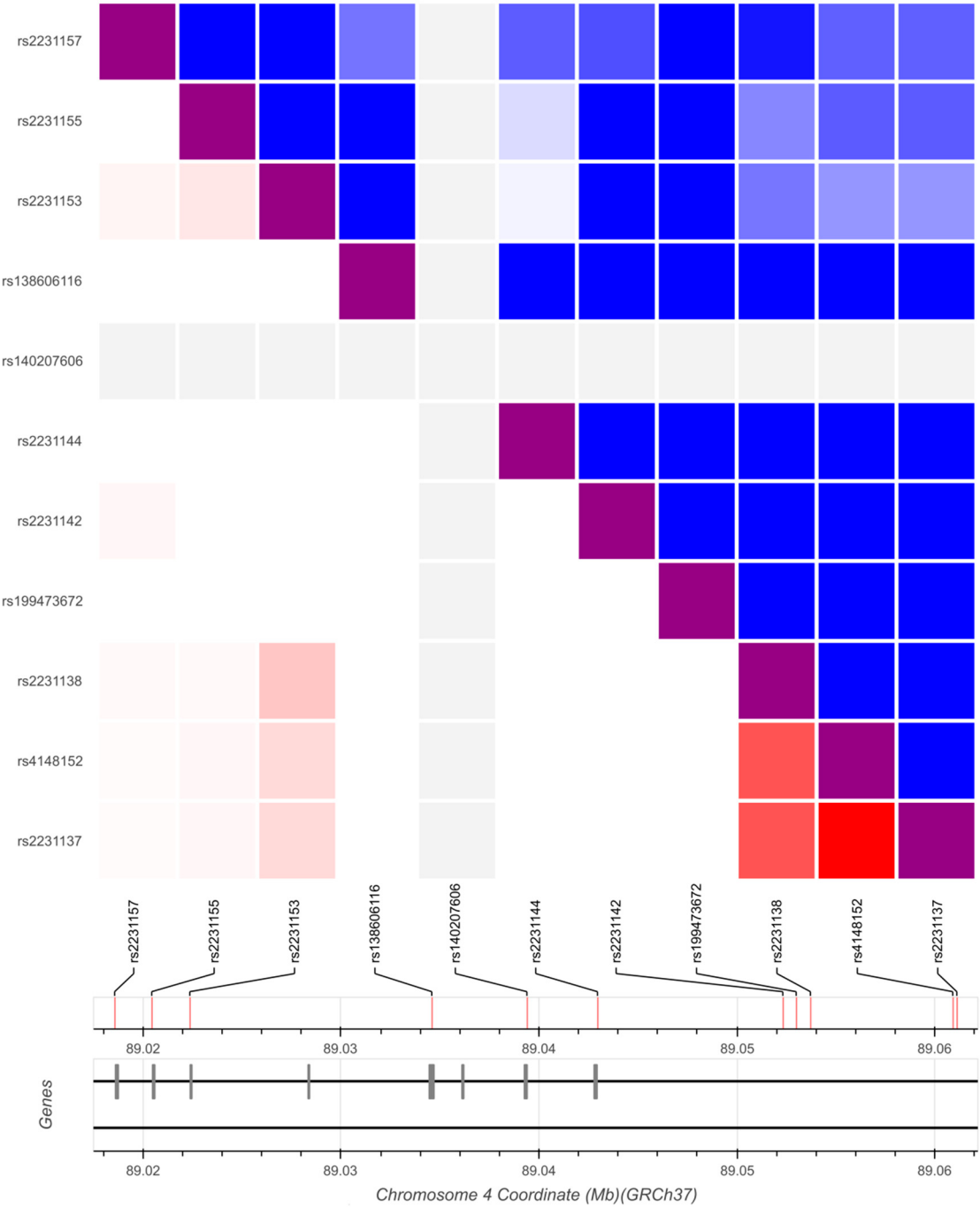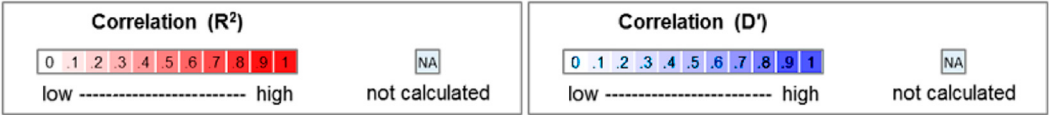

b)

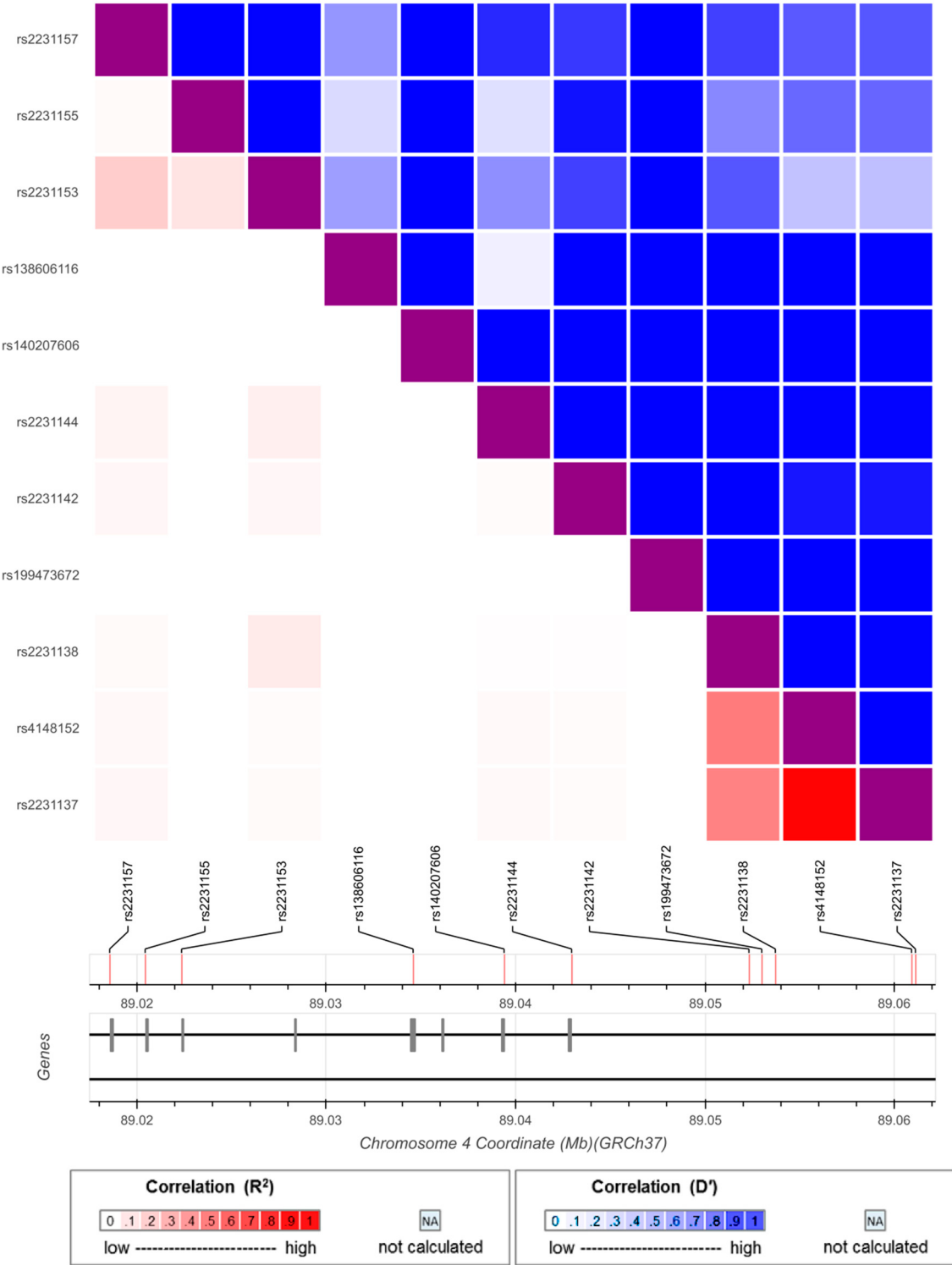

Supplement: Supplementary file 1 [file genes-11-01144-s001.zip › FigS25_LDlink_matrix_plots.pdf]

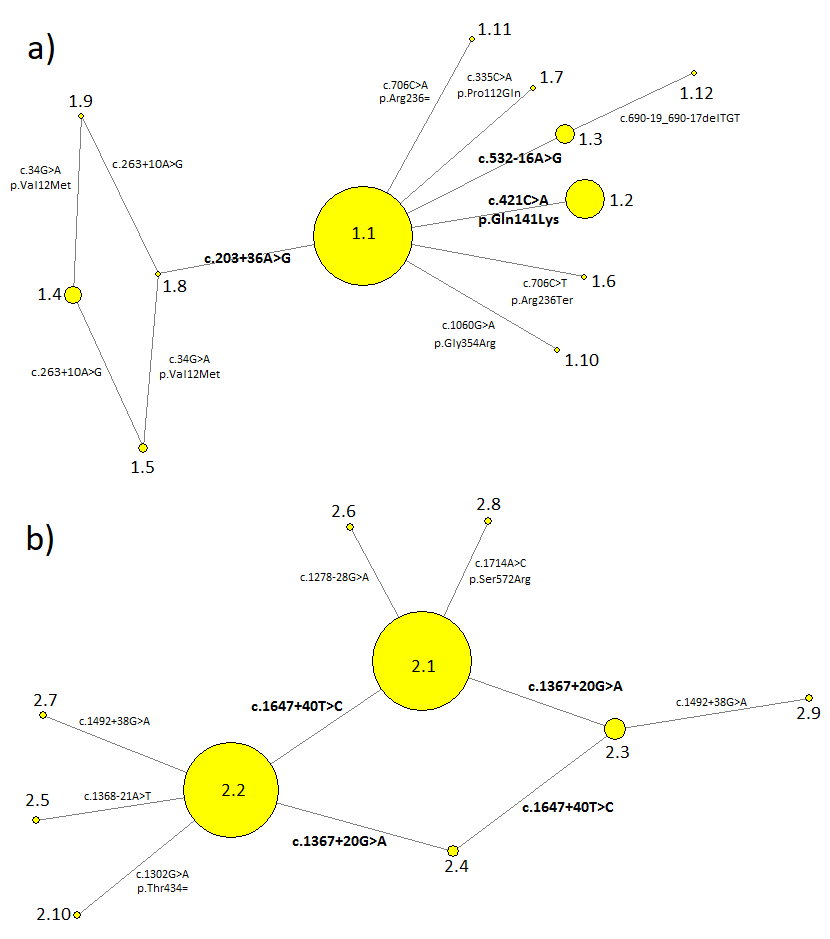

Supplement: Supplementary file 1 [file genes-11-01144-s001.zip › FigS26_Network_block1_block2.png]
